# Lysosomal recruitment of TSC2 is a universal response to cellular stress

**DOI:** 10.1038/ncomms10662

**Published:** 2016-02-12

**Authors:** Constantinos Demetriades, Monika Plescher, Aurelio A. Teleman

**Affiliations:** 1 Division of Signal Transduction in Cancer and Metabolism, German Cancer Research Center (DKFZ), 69120 Heidelberg, Germany

## Abstract

mTORC1 promotes cell growth and is therefore inactivated upon unfavourable growth conditions. Signalling pathways downstream of most cellular stresses converge on TSC1/2, which serves as an integration point that inhibits mTORC1. The TSC1/2 complex was shown to translocate to lysosomes to inactivate mTORC1 in response to two stresses: amino-acid starvation and growth factor removal. Whether other stresses also regulate TSC2 localization is not known. How TSC2 localization responds to combinations of stresses and other stimuli is also unknown. We show that both amino acids and growth factors are required simultaneously to maintain TSC2 cytoplasmic; when one of the two is missing, TSC2 relocalizes to lysosomes. Furthermore, multiple different stresses that inhibit mTORC1 also drive TSC2 lysosomal accumulation. Our findings indicate that lysosomal recruitment of TSC2 is a universal response to stimuli that inactivate mTORC1, and that the presence of any single stress is sufficient to cause TSC2 lysosomal localization.

## Abstract (web summary)

In response to amino acid and growth factor removal the TSC1/2 complex translocates to the lysosome to inactivate mTOR and inhibit cell growth. Here, the authors have shown that other cellular stresses also trigger this translocation to the lysosome suggesting that this is a universal mechanism in the stress response.

## Graphical abstract

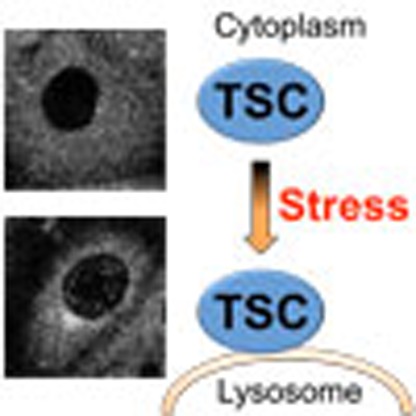

Cells sense their milieu and regulate their growth accordingly. When cells have insufficient nutrients, energy, oxygen, or confront a variety of other stresses, they rewire their metabolism to block anabolic processes and cease growth^1^,^2^,^3^. Mutations in growth-related genes that make cells incapable of adapting to changes in their environment, and thereby promote cell growth even under unfavourable conditions, lead to metabolic catastrophe and ultimately cellular and organismal death^4^,^5^,^6^,^7^,^8^,^9^.

mTOR complex 1 (mTORC1) is a master regulator of cellular growth and metabolism, which is dysregulated in most cancers and in several metabolic disorders^10^,^11^,^12^,^13^. Multiple signals from nutrient availability, growth factor signalling and cellular stresses are integrated to regulate mTORC1 activity^11^,^13^. This occurs via two sets of small GTPases. Most signals regulate the activity of the direct mTORC1 activator Ras homologue enriched in brain (Rheb), whereas amino acids signal to the Rag GTPases to control the localization of mTORC1 to the lysosomal membrane, where Rheb also resides^14^. Hence, to fully activate mTORC1, both requirements must be met: Rheb needs to be in the active state^15^,^16^ and mTORC1 needs to reside in the proximity of Rheb^9^,^14^,^17^.

An important inhibitor of mTORC1 activity is the tuberous sclerosis complex (TSC), which is composed of the TSC1, TSC2 and TBC1D7 proteins^18^. As part of the complex, TSC2 possesses GTPase-activating protein (GAP) activity towards Rheb, catalysing its conversion from the active GTP-bound state to its inactive GDP-bound conformation^15^,^16^. Virtually all upstream stimuli that regulate mTORC1 activity, including amino acids, converge on TSC2 to regulate its function^4^,^19^,^20^,^21^,^22^,^23^,^24^,^25^,^26^,^27^.

The mechanism by which various stresses activate TSC2 is not fully understood. Recent work showed that amino-acid starvation or growth factor removal regulates TSC2 activity in part via its subcellular localization. Upon removal of either amino acids^4^ or insulin signalling^28^,^29^, the TSC1/2 complex is recruited to the lysosome, where mTORC1 is located. This allows TSC2 to inhibit mTORC1 by acting on Rheb, which is in part localized on lysosomes^14^,^29^,^30^,^31^,^32^,^33^,^34^. These observations raise the exciting possibility that regulation of TSC2 subcellular localization might be a universal mechanism by which cellular stresses activate TSC2. Whether this is the case, however, is not known. Also unknown is how TSC2 localization responds to combinations of stresses and other stimuli to integrate this information. For instance, if growth factor stimulation is removed but amino acids are present, is TSC2 cytoplasmic or lysosomal? Finally, although we previously reported that amino acids regulate TSC2 localization, other studies concluded the contrary^29^,^34^. We therefore also investigate here the differences in experimental approaches and cell lines used in these studies, to explain apparent discrepancies in the literature and to understand the underlying biological phenomena.

Here we show that many different stresses lead to lysosomal recruitment of TSC2, including hypoxia, osmostress, energetic stress and pH stress, indicating that lysosomal recruitment of TSC2 is a universal response to a variety of inhibitory stimuli. Furthermore, from different combinations of starvation or stress treatments in an array of diverse cell lines we conclude that each individual stimulus that inhibits mTORC1 is sufficient by itself to cause TSC2 recruitment to the lysosome. Thus, amino-acid starvation relocalizes TSC2 to lysosomes in the presence of serum, and serum deprivation relocalizes TSC2 to lysosomes in the presence of amino acids. Moreover, we identify cell lines that demonstrate aberrant, constitutive lysosomal localization of TSC2, even in the presence of growth factors and nutrients. Together, in addition to providing an explanation for apparent discrepancies in the literature^4^,^29^, these data reveal the integration logic of how TSC2 localization is affected by combinations of stresses and other stimuli, and discover that lysosomal recruitment of TSC2 is a general feature of inhibitory stimuli that inactivate mTORC1.

## Results

### Amino-acid or FBS removal alone is able to relocalize TSC2

We previously showed that removal of amino acids results in the rapid and reversible accumulation of TSC2 on lysosomes, even in the presence of serum, to inhibit mTORC1 (ref. 4). In parallel, others showed that in an analogous fashion TSC2 resides on lysosomal membranes upon growth factor starvation^28^,^29^,^34^ and that this is reversed by re-stimulation with insulin or other growth factors^28^,^29^. To our surprise, two of these studies also concluded that lysosomal localization of TSC2 is not responsive to amino-acid signalling^29^,^34^, based on the observation that exposing cells to two stresses simultaneously by starving them for both serum and amino acids, and then re-adding only amino acids, was not sufficient to cause TSC2 to become cytoplasmic^29^,^34^. Given the different treatment strategies used in these studies, we sought to investigate the subcellular localization of TSC2 in response to amino-acid signalling, growth factor signalling and combinations of the two.

**Figure 1:**
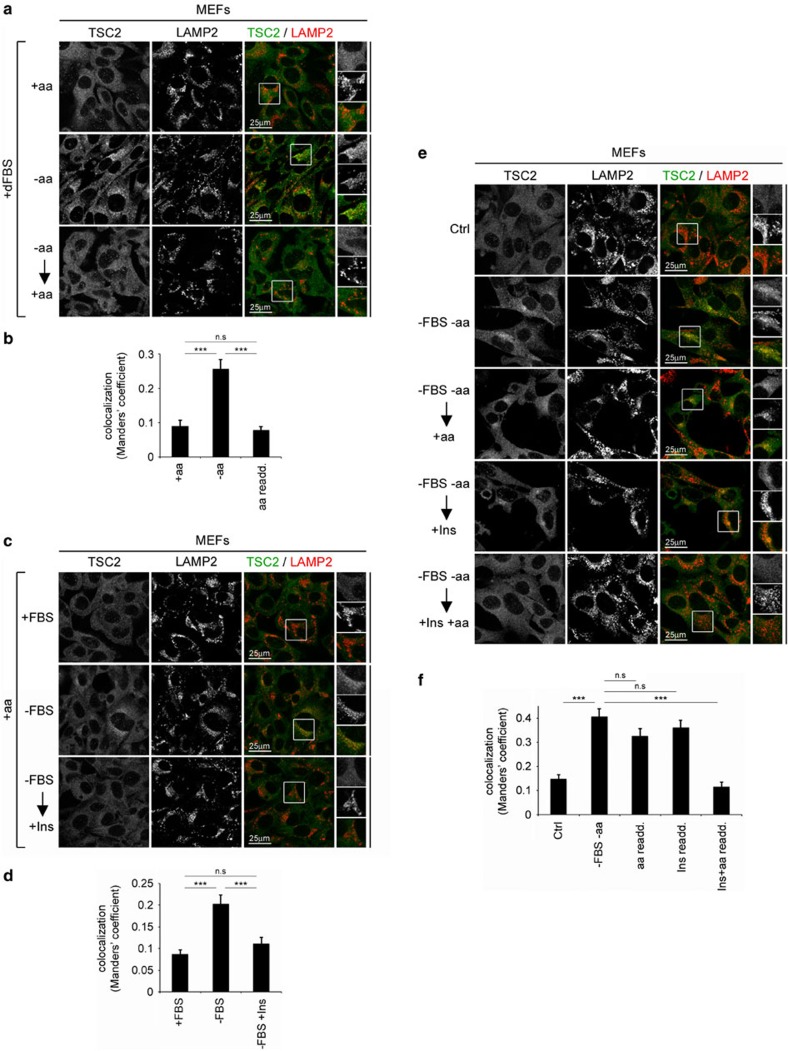
Lack of either amino acids or serum alone is sufficient to cause TSC2 recruitment to lysosomes in MEFs. (**a**,**b**) Amino-acid starvation in the presence of serum causes lysosomal relocalization of TSC2. MEFs were treated with medium containing (+aa) or lacking amino acids (−aa) for 1 h. For re-addition (re-add.) experiments, following 1 h of aa starvation, cells were treated with +aa medium for 30 min. All treatment media contain dFBS (+dFBS). (**a**) TSC2 localization was analysed by immunostaining, and LAMP2 staining was used as a lysosomal marker. Representative magnified insets are shown on the right (top: TSC2; middle: LAMP2; bottom: merged), and the degree of co-localization between TSC2 and LAMP2 (automatically thresholded MCC) is shown in **b**. (**c**,**d**) Serum starvation in the presence of amino acids causes lysosomal relocalization of TSC2. MEFs were treated with medium containing (+FBS) or lacking serum (−FBS) for 16 h. For insulin treatments, cells were starved for 16 h from FBS and then treated with 1 μM insulin for 15 min before fixation. Quantification in **d** was performed as in **b**. (**e**,**f**) Re-addition of amino acids or insulin alone in doubly starved cells is not sufficient to reverse TSC2 lysosomal localization. MEFs were starved for both FBS (16 h) and amino acids (1 h), and then either insulin (1 μM, 15 min) or +aa media (30 min), or both were added back to the cells. Note that the presence of both amino-acid and growth factor signalling is necessary to abrogate lysosomal accumulation of TSC2 and that absence of either one is enough to cause TSC2 lysosomal recruitment. Quantification in **f** was performed as in **b**. For all panels, images representative of at least three independent biological replicates are shown. Quantification of co-localization is shown as mean±s.e.m. ***P<0.001 comparing samples as indicated, using one-way ANOVA. See also Supplementary Figs 1 and 2.

Using mouse embryonic fibroblasts (MEFs), we first tested the effect of amino-acid signalling on TSC2 localization (Fig. 1a,b) and confirmed our previous result that amino-acid removal causes lysosomal recruitment of TSC2 (ref. 4): Upon treatment of MEFs with medium lacking only amino acids, in the presence of dialysed fetal bovine serum (FBS), TSC2 accumulates on lysosomes (marked by LAMP2) (Fig. 1a, middle). In contrast, under basal conditions, cells treated with amino-acid-replete medium demonstrate a more diffuse, cytoplasmic TSC2 signal, which does not show significant accumulation on lysosomal areas (Fig. 1a, top). The lysosomal recruitment of TSC2 is reversible, as re-addition of amino-acid-containing media to cells starved for amino acids rapidly delocalized TSC2 from lysosomes (Fig. 1a, bottom). As a control, amino-acid removal inactivated mTORC1 (observed as a reduction in the phosphorylation of its direct substrate S6 kinase, S6K), and re-addition of amino acids to starved cells rapidly restored mTORC1 activity, as expected (Supplementary Fig. 1a). Next, we tested the effect of growth factor signalling on TSC2 localization and reproduced the observations of others that growth factor deprivation causes lysosomal recruitment of TSC2 (refs 29, 34): MEFs starved overnight of serum, in the presence of amino acids, showed lysosomal accumulation of TSC2, which was reversed by acute treatment with insulin, shortly before fixation (Fig. 1c,d). As controls, insulin stimulation or FBS re-supplementation potently rescued mTORC1 activity in serum-starved MEFs, as expected (Supplementary Fig. 1b). Put together, these results indicate that removal of either amino acids or serum alone is sufficient to induce the lysosomal relocalization of TSC2, even in the presence of serum or amino acids, respectively.

We next tested how combinations of amino-acid signalling and growth factor signalling integrate to regulate TSC2 localization. We starved MEFs for both serum and amino acids and assayed TSC2 localization upon re-stimulation either with amino acids or with growth factors singly. Starvation of cells for both FBS and amino acids leads to a strong lysosomal accumulation of TSC2 (Fig. 1e,f). Adding back either amino acids or insulin alone to doubly starved MEFs mildly reduced but did not abolish the lysosomal accumulation of TSC2 (Fig. 1e,f). In contrast, re-addition of medium containing both amino acids and insulin restored TSC2 localization back to the cytoplasm (Fig. 1e,f). These data indicate that if cells are exposed to two stresses simultaneously (amino-acid removal and growth factor deprivation), reverting only one of the two stresses is not sufficient to revert the lysosomal localization of TSC2. This is in agreement with the data in Fig. 1a,c showing that each stress alone is able to induce lysosomal localization of TSC2. These data paralleled the combinatorial effects of serum starvation and amino-acid removal on mTORC1 activity. Re-addition of only amino acids or only growth factors (dialysed FBS; dFBS) to doubly starved cells only mildly reactivated mTORC1, whereas re-addition of both caused strong mTORC1 reactivation (Supplementary Fig. 1c). Specificity of the α-TSC2 antibody used in this study in immunofluorescence experiments was verified by comparing the TSC2 signal in wild-type (WT) and *TSC2*-null MEFs (Supplementary Fig. 2), in agreement with previous reports^4^,^18^. In sum, either absence of amino acids (in the presence of physiological levels of insulin signalling) or absence of growth factor signalling (in the presence of physiological levels of amino acids) is sufficient to drive accumulation of TSC2 on lysosomes. The presence of both amino acids and insulin is necessary to keep TSC2 cytoplasmic.

### Starvation induces TSC2 relocalization in diverse cell lines

**Figure 2:**
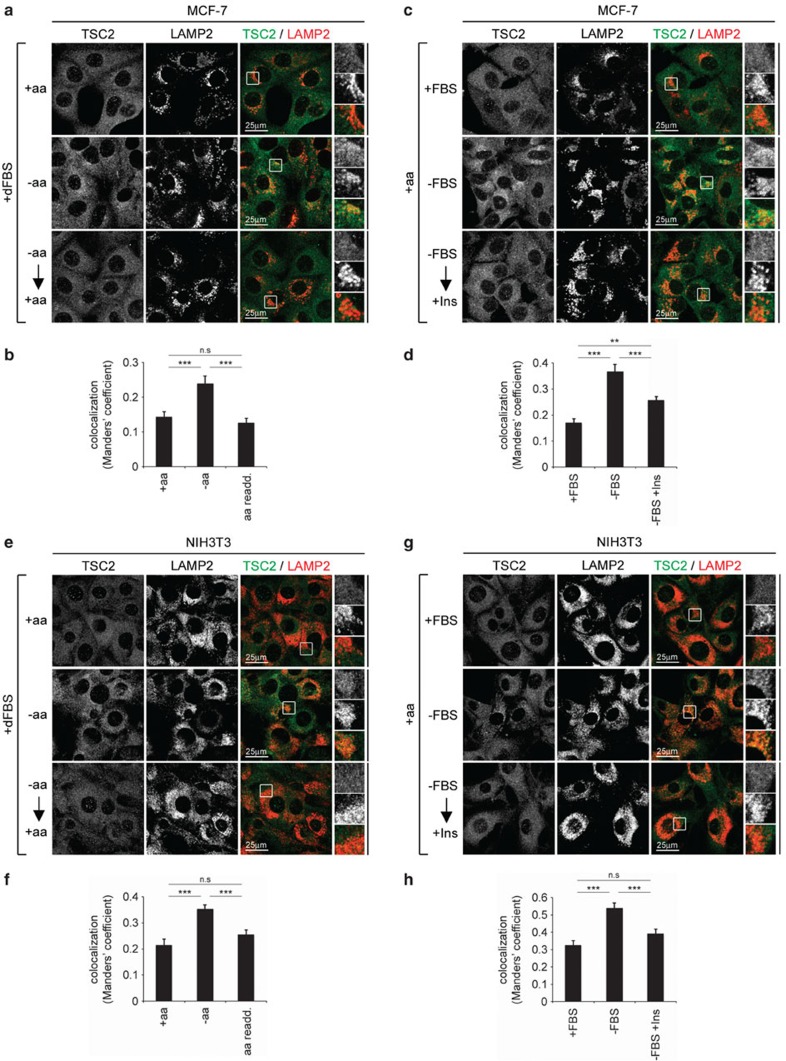
Lack of amino acids or serum alone is sufficient to relocalize TSC2 to lysosomes in different cell lines. (**a**–**d**) Amino-acid or serum starvation causes lysosomal relocalization of TSC2 in breast adenocarcinoma MCF-7 cells. Treatments to assay the effects of amino-acid starvation and re-addition (**a**,**b**) or the effects of serum starvation and insulin re-stimulation (**c**,**d**) on TSC2 localization in MCF-7 cells were performed as in Fig. 1a,c, respectively (representative of three biological replicates). (**e**–**h**) Same as in **a**–**d**, using MEF NIH3T3 cells (representative of two biological replicates). For all panels, the degree of co-localization between TSC2 and LAMP2 (automatically thresholded MCC) is shown as mean±s.e.m. ***P<0.001, **P<0.01 comparing samples as indicated, using one-way ANOVA. See also Supplementary Figs 3–5. re-add., re-addition.

We next tested whether the effects described above are specific for MEFs, or not. We performed amino-acid and serum starvation experiments on a wide array of established cell lines of different origins, such as human breast adenocarcinoma MCF-7 (Fig. 2a–d and immunoblot controls in Supplementary Fig. 3a,b), MEF NIH3T3 (Fig. 2e–h and immunoblot controls in Supplementary Fig. 3c,d), human embryonic kidney HEK293FT (Supplementary Fig. 4a,b,d,e and immunoblot controls in Supplementary Fig. 4c,f) and mouse hepatoma Hepa1-6 cells (Supplementary Fig. 5a,b,d,e and immunoblot controls in Supplementary Fig. 5c,f). In MCF-7 and NIH3T3 cells, TSC2 is diffusely cytoplasmic in medium containing amino acids, becomes concentrated on lysosomes upon amino-acid removal, and returns to a diffuse cytoplasmic localization upon amino-acid re-addition (Fig. 2a,e). Similarly, in these cell lines, serum starvation induced lysosomal accumulation of TSC2, which was reversed by insulin stimulation back to the uniform cytoplasmic distribution also observed in control conditions (Fig. 2c,g). In a similar fashion, TSC2 relocalized on amino-acid removal or serum starvation in HEK293FT and Hepa1-6 cells, although to a milder extent (Supplementary Figs 4 and 5). These experiments confirm the results obtained from MEFs (Fig. 1a,c) and show that TSC2 relocalization to the lysosome upon either amino-acid or serum starvation is a general phenomenon, observed in a variety of different cell types.

### TSC relocalizes in response to multiple different stresses

**Figure 3:**
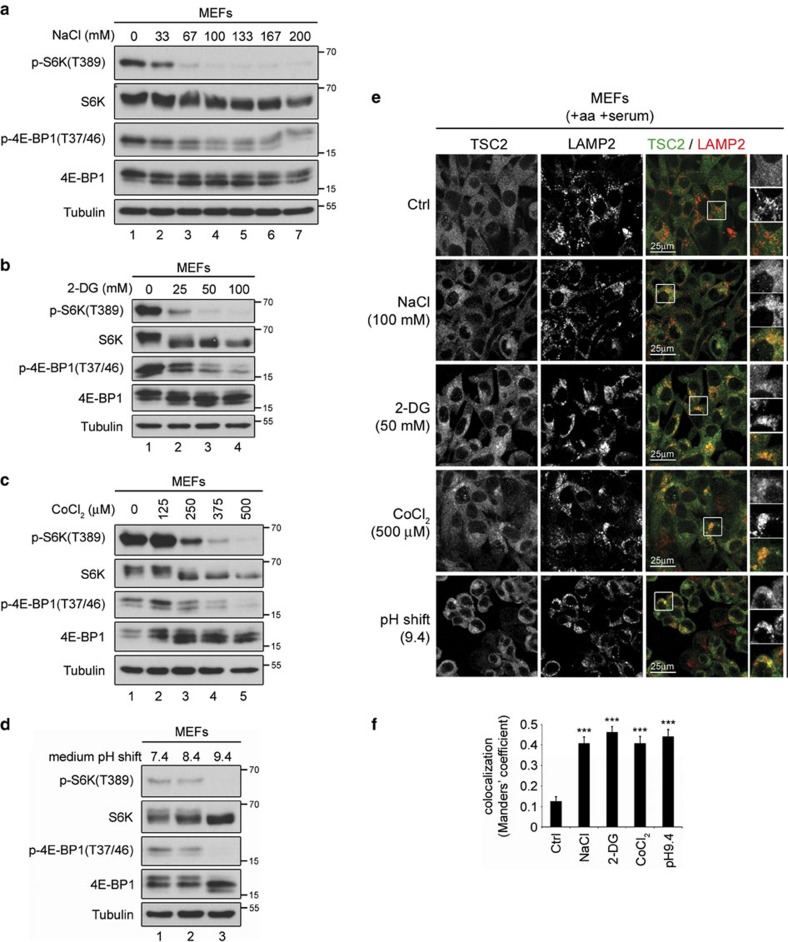
Many different stress stimuli that inactivate mTORC1 cause TSC2 to relocalize to lysosomes. (**a**) Hyperosmotic stress inactivates mTORC1 in a dose-dependent manner. MEFs were exposed to normal or hyperosmotic culture media for 15 min. Osmolality of the medium was elevated by increasing NaCl concentration by 33–200 mM above baseline. (**b**) Energetic stress inactivates mTORC1 in a dose-dependent manner. MEFs were treated with the indicated amounts of 2-DG for 30 min. (**c**) Hypoxic stress inactivates mTORC1 in a dose-dependent manner. MEFs were treated for 24 h with the indicated amounts of CoCl_2_, a compound which partially mimics hypoxia. (**d**) mTORC1 is inactivated by changes in the pH of the culture media. MEFs were treated with pH-adjusted media for 30 min. (**e**,**f**) TSC2 relocalizes to lysosomes upon exposure of cells to all the stresses tested singly. MEFs were cultured in full medium (+aa +serum). Cells were exposed to each stress as indicated in the figure and as described in **a**–**d**, and TSC2 localization was analysed by immunostaining. LAMP2 staining was used as a lysosomal marker. Representative magnified insets are shown on the right (top: TSC2; middle: LAMP2; bottom: merged). The degree of co-localization between TSC2 and LAMP2 (automatically thresholded MCC) is shown in **f** as a mean±s.e.m. ***P<0.001 for comparison to control (Ctrl), using one-way ANOVA. Note that each stress stimulus is sufficient to cause lysosomal recruitment of TSC2 in the presence of serum and amino acids. For all treatments, data representative of at least three independent biological replicates are shown. See also Supplementary Figs 6–10.

**Figure 4:**
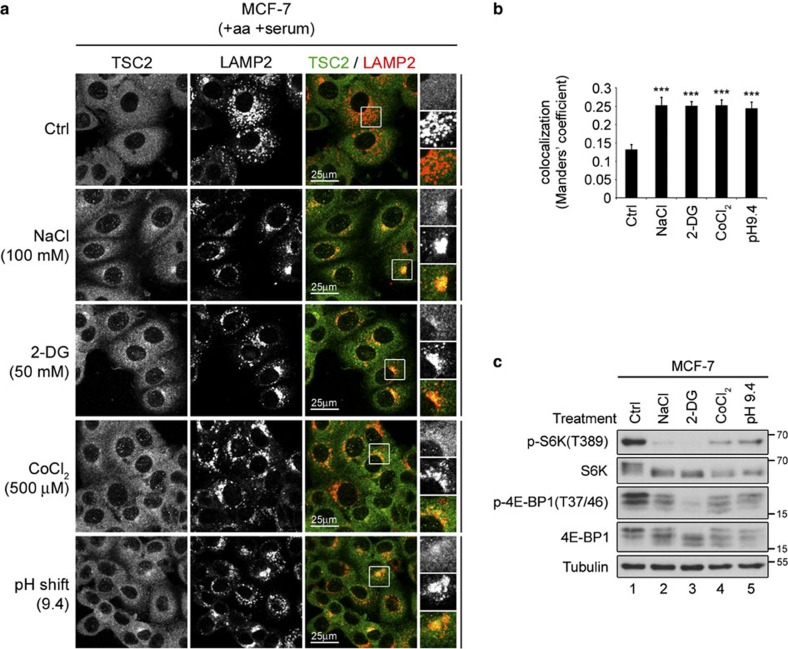
The lysosomal relocalization of TSC2 upon stresses is not cell-type specific. (**a**,**b**) The lysosomal accumulation of TSC2 upon stresses is not specific to MEFs. Breast adenocarcinoma MCF-7 cells were treated as in Fig. 3e, and TSC2 localization was analysed by immunofluorescence. LAMP2 staining was used as a lysosomal marker. Representative magnified insets are shown on the right (top: TSC2; middle: LAMP2; bottom: merged), and co-localization was quantified in **b** as in Fig. 3f (shown as mean±s.e.m.). Note that each individual inhibitory stimulus is sufficient to cause lysosomal relocalization of TSC2, when applied singly to MCF-7 cells grown in serum- and amino-acid-replete media. ***P<0.001 compared with control (Ctrl), using one-way ANOVA. (**c**) Individual stresses inactivate mTORC1 in MCF-7 cells. Cells were treated as in **a**, and mTORC1 activity was analysed by immunoblotting with the indicated antibodies. For all panels, data representative of three independent biological replicates are shown. See also Supplementary Fig. 7.

**Figure 5:**
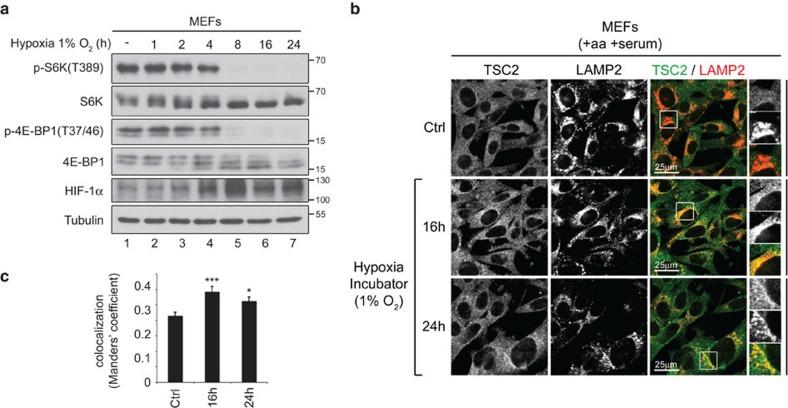
Hypoxia inhibits mTORC1 and causes lysosomal accumulation of TSC2. (**a**) Hypoxia inactivates mTORC1. MEFs were incubated in a hypoxic chamber (1% O_2_) for the indicated times before lysis, and mTORC1 activity was assayed by immunoblot using the indicated antibodies (representative of three independent biological replicates). (**b**,**c**) TSC2 relocalizes to lysosomes upon hypoxia. MEFs were incubated in a hypoxic chamber (1% O_2_) for the indicated times before fixation, and TSC2 localization was assayed by immunofluorescence. Representative magnified insets are shown on the right (top: TSC2; middle: LAMP2; bottom: merged), and quantification in **c** was performed as in Fig. 3e,f (shown as mean±s.e.m.). ***P<0.001, *P<0.05 for comparison with control (Ctrl) cells, using one-way ANOVA (representative of two independent biological replicates).

Because serum or amino-acid starvation alone—two conditions that lead to mTORC1 inactivation—was sufficient to cause recruitment of TSC2 to lysosomes, we reasoned that other stimuli that inhibit mTORC1 might also have the same effect on TSC2 localization. We therefore tested five stress conditions that cause mTORC1 inactivation when applied to cells: hyperosmotic stress; energetic stress; pH stress; hypoxia; and cobalt chloride, which partially but not completely phenocopies hypoxia^9^,^20^,^21^,^22^,^23^,^24^,^35^,^36^,^37^,^38^,^39^. For this purpose, we treated MEFs with increasing concentrations of sodium chloride (NaCl), 2-deoxy-D-glucose (2-DG), cobalt chloride (CoCl_2_) or pH-adjusted media, respectively, to identify the minimal treatment conditions that are able to robustly drop mTORC1 activity (Fig. 3a–d). Subsequently, we assayed TSC2 localization on control or stress conditions. Interestingly, each single stress stimulus caused strong lysosomal accumulation of TSC2 in MEFs (Fig. 3e,f) or MCF-7 cells (Fig. 4a,b and immunoblot controls in Fig. 4c), despite the presence of both amino acids and serum in the culture media. Similarly, incubation of cells in a hypoxic chamber (1% O_2_) also caused mTORC1 inactivation (Fig. 5a) and TSC2 relocalization to lysosomes (Fig. 5b,c). Furthermore, consistent with 2-DG blocking glycolysis by competing with glucose in the growth medium, significantly lower concentrations of 2-DG were able to both robustly inhibit mTORC1 (Supplementary Fig. 6a), and induce TSC2 lysosomal relocalization (Supplementary Fig. 6b,c) when cells were first incubated in low-glucose medium.

All stimuli that inhibit mTORC1 and induce accumulation of TSC2 on lysosomes also lead to a similar lysosomal relocalization of TSC1 in both MEFs (Supplementary Fig. 7a,b) and MCF-7 cells (Supplementary Fig. 7c,d). This extends our previous observation that both TSC2 and TSC1 relocalize to lysosomes upon amino-acid starvation^4^. As with the α-TSC2 antibody, the specificity of the α-TSC1 antibody in these experiments was confirmed by comparing the TSC1 signal in WT and *TSC1*-null MEFs (Supplementary Fig. 7e).

Previous work suggested that TSC2 relocalizes to peroxisomes in response to high levels of reactive oxygen species (ROS)^40^. We could detect little or no co-localization of TSC2 with the peroxisomal marker PMP70 in response to the various stresses tested in this study (Supplementary Fig. 8a,b), indicating that lysosomes, and not peroxisomes, are the predominant organelles to which the TSC complex is targeted in response to these stress stimuli in the cells we tested.

To complement the immunostaining approaches, we previously showed that TSC2 relocalizes to the lysosomal surface upon amino-acid removal by immunoelectron microscopy, and by detecting increased binding of TSC2 to the Rag GTPases, which are lysosomally localized^4^. Likewise, we find increased binding of TSC2, TSC1 and TBC1D7 to the Rag GTPases in response to various stresses (Supplementary Fig. 9), in agreement with relocalization of the entire TSC complex to lysosomes. Consistent with previous reports^4^,^19^,^20^,^21^,^22^,^23^,^24^,^27^, TSC2 was required for complete mTORC1 inhibition in response to all of these stresses (Supplementary Fig. 10).

In sum, these data indicate that a wide array of stresses that inhibit mTORC1 each cause lysosomal accumulation of the TSC complex when applied singly to cells, and that TSC2 and TSC1 are cytoplasmic only when cells experience none of these stresses.

### HeLa cells have constitutive lysosomal accumulation of TSC2

**Figure 6:**
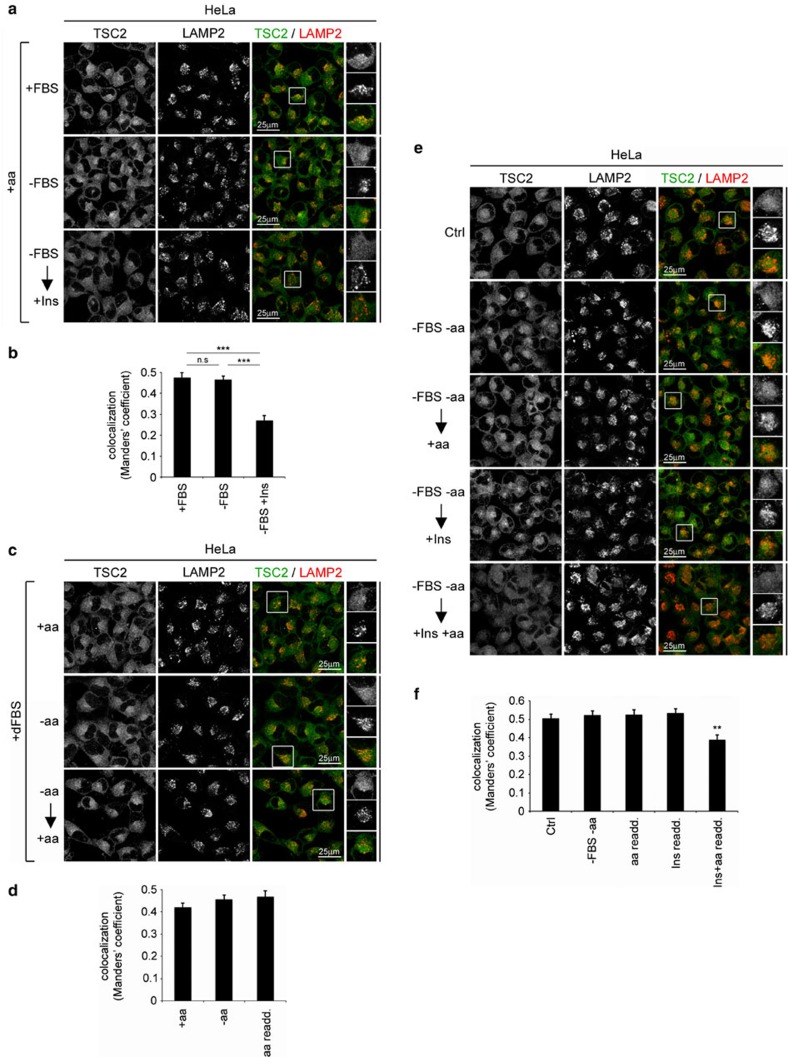
HeLa cells have constitutive lysosomal TSC2 localization even under basal culture conditions. (**a**,**b**) In HeLa cells, TSC2 is concentrated on lysosomes in basal, serum-containing conditions and only relocalizes to the cytoplasm upon hyperstimulation with insulin (1 μM). (**c**,**d**) In HeLa cells, TSC2 constitutively accumulates on lysosomes in medium containing serum and amino acids. Since amino-acid levels present in DMEM are not sufficient to dislodge TSC2 from lysosomes in HeLa cells, TSC2 is lysosomally accumulated both in basal culture conditions (top row, +aa), and when amino acids are first removed and then restored to the starting concentrations (bottom row). (**e**,**f**) The re-addition (re-add.) of both amino acids and high insulin are required to blunt TSC2 lysosomal localization in doubly starved HeLa cells. HeLa cells were starved for both FBS (16 h) and amino acids (1 h), and then either insulin (1 μM, 15 min), +aa media (30 min) or both were added back to the cells. Note the presence of lysosomal TSC2 even upon high insulin re-stimulation (−FBS −aa→+Ins), which is only blunted when amino acids are also added back, indicating that both insulin signalling and amino acids are required for cytoplasmic localization of TSC2. For **a**,**c** and **e** TSC2 localization was assayed in HeLa cells as in Fig. 1, and the degree of co-localization between TSC2 and LAMP2 (automatically thresholded MCC) is shown in **b**,**d** and **f** as mean±s.e.m. ***P<0.001, **P<0.01 for comparison to control (Ctrl) or as indicated, using one-way ANOVA. For all panels, images representative of at least three independent biological replicates are shown. See also Supplementary Figs 11–16.

In HeLa cells, as previously described^29^, and as observed here for other cell lines, TSC2 also accumulates on lysosomes upon serum starvation and relocalizes to the cytoplasm upon re-stimulation with insulin (Fig. 6a, middle and bottom rows). Unexpectedly, however, HeLa cells showed significant lysosomal TSC2 accumulation even in basal conditions in the presence of amino acids and serum (Fig. 6a–e, top row). This is in stark contrast to the cell lines analysed above, and can also be observed as significantly higher TSC2/LAMP2 co-localization coefficients in HeLa cells (circa 0.4) compared with other cell lines such as MEFs, MCF-7 or HEK293FT cells (circa 0.15). This constitutive lysosomal localization of TSC2 indicates that the levels of growth factors and amino acids present in standard culture medium are not sufficient to delocalize TSC2 away from lysosomes in HeLa cells, and that hyperstimulation with insulin is required to dislodge TSC2 from lysosomes. In agreement with this, a previous report showed that significant numbers of serum-starved HeLa cells maintain lysosomal localization of TSC2 upon insulin re-stimulation unless high levels of insulin (1 μM, as in Fig. 6a) are used (Supplementary Fig. S2F in ref. 29). The constitutive lysosomal localization of TSC2 under basal conditions also influences the result of amino-acid removal and re-addition: Since TSC2 is already lysosomally accumulated in basal conditions (Fig. 6c, top row), amino-acid removal had little or no additional effect on its localization in HeLa cells (Fig. 6c, middle row; and Fig. 6d). Moreover, since the levels of amino acids in culture medium are not sufficient to dislodge TSC2 from lysosomes in HeLa cells (Fig. 6a,c,e, top row), TSC2 remained lysosomally localized when amino acids were returned to the starting levels of culture medium (Fig. 6c, bottom row; quantification in Fig. 6d; and western blot controls in Supplementary Fig. 11).

We next studied the combinatorial effect of insulin and amino-acid signalling on TSC2 localization in HeLa cells (Fig. 6e,f). Simultaneous removal of both serum and amino acids in HeLa cells causes a mild, nonsignificant enrichment of TSC2 on lysosomes compared with the basal condition (Fig. 6e, top two rows; and Fig. 6f). Neither add-back of high insulin levels alone nor of amino acids alone was able to strongly dislodge TSC2 from lysosomes (Fig. 6e, rows 3 and 4; and Fig. 6d). In contrast, hyperstimulation with 1 μM insulin combined with add-back of amino acids was required to significantly reduce the lysosomal accumulation of TSC2 (Fig. 6e, bottom row; and Fig. 6f). These findings are consistent with the results presented above for all other cell lines that both amino-acid signalling and insulin signalling are required for cytoplasmic localization of TSC2, compounded with the fact that HeLa cells have strong constitutive lysosomal accumulation of TSC2.

The identity of our HeLa batch was verified using the Multiplex Cell Line Authentication service by Multiplexion GmbH, which uses a single-nucleotide polymorphism (SNP)-profiling approach. Furthermore, we confirmed our results using an independent batch of HeLa cells, obtained from the American Type Culture Collection (ATCC) repository (Supplementary Fig. 12a,b). Since we supplement the treatment media that we use in amino-acid starvation and re-addition experiments with dFBS (to remove amino acids), we also tested whether the FBS dialysis might be causing the constitutive recruitment of TSC2 to lysosomes. This was not the case: HeLa cells treated with full or dialyzed FBS in the presence of amino acids showed an indistinguishable accumulation of TSC2 localization on lysosomes (Supplementary Fig. 12c,d). In sum, we conclude that HeLa cells are unlike other cells, in that they have constitutive, lysosomally localized TSC2, even in the presence of amino acids and serum, which only delocalizes to the cytoplasm on hyperstimulation.

We next tested the effects of cellular stresses on TSC2 localization in HeLa cells. Since HeLa cells show lysosomal accumulation of TSC2 even in basal, non-stressed conditions, stresses did not have a strong additional effect on TSC2 localization in these cells (Supplementary Fig. 13a,b and immunoblot controls in Supplementary Fig. 13c,d). Interestingly, despite having constitutive TSC2 localization under control conditions, HeLa cells grow well in culture and demonstrate similar mTORC1 activity, compared with MEFs (Supplementary Fig. 14a). In addition to other abnormalities that are known to exist in HeLa cells, they are known not to express LKB1 (refs 41, 42), which is implicated in mTORC1 signalling by regulating AMPK-mediated TSC2 phosphorylation^43^,^44^. We therefore tested whether exogenous expression of LKB1 in HeLa cells is able to rescue the irregular TSC2 and TSC1 localization in basal conditions, but this was not the case (Supplementary Fig. 14b–f). Nonetheless, the fact that HeLa cells have comparable levels of mTORC1 activity as MEFs, despite constitutively lysosomal TSC2, suggests HeLa cells have other compensatory aberrations that promote high mTORC1 activity.

We searched for other cell lines that have constitutive lysosomal accumulation of TSC2 under standard cell culture conditions, and found two: African green monkey (*Cercopithecus aethiops*) kidney fibroblast-like COS cells; and human liver hepatocellular carcinoma HepG2 cells. Interestingly, in these cells TSC2 was lysosomally concentrated in all conditions—in standard growth medium, when starved of amino acids or FBS, and even when hyperstimulated with high levels of insulin (Supplementary Figs 15 and 16). Thus, the relocalization of TSC2 in COS and HepG2 cells in response to various stimuli is completely aberrant, whereas HeLa cells are only partially defective.

## Discussion

TSC2 is an important inhibitor of mTORC1 activity. We previously showed that in response to amino-acid starvation, TSC2 accumulates on lysosomal surfaces to act on Rheb, the direct mTORC1 activator, thereby inhibiting mTORC1 (ref. 4). Although the role of amino acids in the regulation of mTORC1 localization and activity is well established^45^,^46^,^47^,^48^, whether amino acids also regulate TSC2 localization has been debated^4^,^29^,^34^. Here we confirm and extend our previous findings to show that amino acids do regulate TSC2 localization, and do so in several different cell types. In addition, by studying the effect of other stresses on TSC2 localization, such as hyperosmotic stress, energetic stress, hypoxia and changes in pH, we find that all of them affect TSC2 localization (for hyperosmotic stress, see also ref. 49). This suggests that regulation of TSC2 localization is a universal mechanism by which stresses regulate TSC2. This also highlights the notion that the regulation of TSC2 subcellular localization is not specific of insulin signalling.

**Figure 7:**
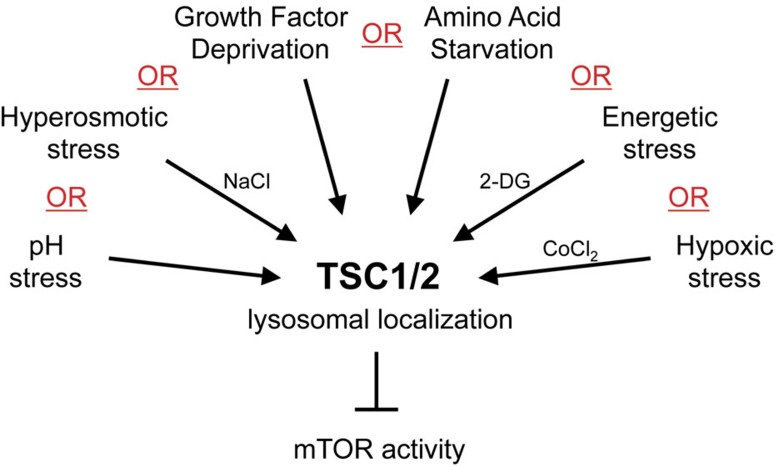
Recruitment of TSC2 to lysosomes is a universal feature of inhibitory stimuli towards mTORC1 inactivation. Schematic model depicting the relationship between various stimuli that inhibit mTORC1 and their effect on lysosomal relocalization of TSC2. Each individual stress or starvation condition alone is sufficient to induce recruitment of TSC2 to lysosomes and thereby to inhibit mTORC1. Only when all stresses are absent is TSC2 cytoplasmic. The integration of all inhibitory stimuli on TSC2 ensures that mTORC1 will be inactivated in all situations, where conditions are not optimal and therefore do not favour growth.

We analyse here the combinatorial effect of different stresses on TSC2 localization. It is well described that both amino-acid and growth factor signalling are necessary simultaneously to fully activate mTORC1, and that each stimulus alone is not sufficient^13^,^46^,^50^. Furthermore, various different stress signals are known to inhibit mTORC1, also in the presence of nutrients and growth factors^19^. In sum, mTORC1 is only active when multiple conditions are simultaneously met—amino acids are sufficient, growth factor signalling is present, cells do not confront any stress stimuli and so on. If any one of these conditions is not met, mTORC1 is inactivated. From the mathematical point of view, the Boolean operator used by mTORC1 to ‘decide' whether to be active is the ‘OR' operator. If amino acids are missing, OR growth factors are missing, OR cells are hypoxic, OR cells have low energy, OR any other cellular stress is present, then mTORC1 is off. From the biological point of view, this ensures that cells will inactivate mTORC1 and therefore cease growth on any condition that is not optimal. In the same way, we find that TSC2 accumulates on lysosomes to inactivate mTORC1 if any one of these conditions is met (Fig. 7). This makes sense since TSC2 is an integration point, where all the various stresses converge to regulate mTORC1. Each stress alone causes TSC2 to accumulate on lysosomes. If two stresses are applied simultaneously to cells, as in the case of double starvation for serum and amino acids, then both stresses need to be relieved for TSC2 to return to the cytoplasm. If only one stress is relieved, and the other is maintained (for example, if cells are starved of FBS and amino acids, and then only re-stimulated with amino acids), then TSC2 remains lysosomal to inactivate mTORC1. This in part explains misinterpretations in the literature regarding whether amino acids regulate TSC2 localization using experimental set-ups, whereby multiple stresses were applied simultaneously to cells, but only one stress was relieved by an add-back experiment^29^,^34^. For instance, when HCT-116 colorectal carcinoma cells were starved for both amino acids and serum, and then re-stimulated with only amino acids, they retained lysosomal TSC2 localization (Fig. 8 in ref. 34). This is not because amino acids do not regulate TSC2 localization, but because the serum deprivation stress is still present, in agreement with the findings reported here (Figs 1 and 2 and Supplementary Figs 4 and 5). The work presented here points out the importance of carefully selecting the appropriate treatment strategies when working with signalling pathways such as mTOR that are influenced by multiple upstream stimuli and integrate information. Along with previous studies^4^,^29^,^51^, our results indicate that lysosomal localization of the TSC complex is a shared feature of signals that inhibit mTORC1.

HeLa cells are known to be aberrant in multiple ways, even when compared with other cancer-derived cell lines^52^. They are aneuploid, with a hyper-triploid chromosome number, show inherent genomic instability with extensive chromothripsis and lack expression of important genes, including *LKB1*, which is involved in mTORC1 signalling^41^,^42^,^53^,^54^,^55^,^56^. Likewise, we find that HeLa cells also have an aberrant constitutive accumulation of TSC2 on lysosomes that is only blunted by hyperstimulation with super-physiological levels of insulin. Hence, in HeLa cells growing in complete Dulbecco's modified Eagle's medium (DMEM), which contains both serum and amino acids, TSC2 is lysosomally accumulated. When amino acids are removed, and then returned to the starting DMEM levels, TSC2 is still lysosomally concentrated just as in complete DMEM. This has previously been interpreted to mean that amino acids do not affect TSC2 localization (Fig. 4e in ref. 29), however the basal starting condition was omitted in this study, and the results reflect a peculiarity of HeLa cells. Indeed, when HeLa cells are starved of both serum and amino acids, re-addition of only super-physiological levels of insulin is not sufficient to completely relocalize TSC2 to the cytoplasm (Fig. 6e,f). Instead, amino acids also need to be resupplied to strongly relocalize TSC2 to the cytoplasm (Fig. 6e, bottom row; and Fig. 6f) indicating that amino acids are indeed needed for cytoplasmic localization of TSC2 also in HeLa cells. We tested whether the loss of *LKB1* in HeLa cells is responsible for this peculiarity, but exogenous LKB1 expression was not sufficient to rescue the irregular TSC1/2 localization (Supplementary Fig. 14b–f). The constitutively lysosomal accumulation of TSC2 and TSC1 in HeLa cells suggests these cells have modified signalling properties influencing TSC1/2 localization. It should be noted, however, that in HeLa cells, mTORC1 is active under basal conditions, and mTORC1 activity responds reasonably well to stresses. Hence, there are likely additional, localization-independent layers of regulation of TSC2 activity remaining to be discovered, which are functional in cells, including HeLa cells. Nonetheless, previous work has clearly shown that the subcellular localization of TSC2 is one important aspect regulating TSC2 activity^29^.

We show here that TSC2 relocalizes to lysosomes either upon starvation conditions or in response to multiple individual stresses. The molecular mechanisms controlling TSC2 localization remain largely unknown and represent an interesting direction for future research. TSC2 is a heavily phosphorylated protein that integrates signals from several upstream pathways^19^,^57^. Consistent with this, TSC2 localization on membranes is controlled by Akt-mediated phosphorylation of TSC2 in response to growth factor signalling^28^,^29^. Since Akt activity is not affected upon short-term amino-acid starvation^46^,^58^,^59^, it is possible that other phosphorylation changes on TSC2 might be regulating its localization in response to amino acids or the other stresses studied here. Indeed, although all the various stresses we tested cause TSC2 to relocalize to lysosomes, we believe it is likely that each stress does so via a different mechanism. For instance, treatment of cells with 2-DG or pH 9.4 medium causes a stronger increase in TSC2-Rag GTPase binding compared with the other stresses. Thus, although relocalization of TSC2 appears to be a universal response to cellular stress, the molecular mechanism by which this happens may vary from stress to stress.

In most cell lines we tested, in addition to the TSC2 accumulations that co-localize with LAMP2, we also observe some accumulations of TSC2 that do not co-localize with this lysosomal marker, suggesting they are not lysosomal. Both Rheb and mTOR have previously been described to localize not only to lysosomes/late endosomes but also to other organelles such as the Golgi and mitochondria^14^,^30^,^31^,^32^,^33^,^60^. In addition, recent reports have shown that under particular circumstances TSC2 can also be found on the Golgi^61^ or on peroxisomes^40^. Therefore, it is possible that, upon inhibitory stimuli, TSC2 relocalizes not only to lysosomes but to a lesser extent also to other subcellular compartments containing mTORC1 to inhibit Rheb.

Summarizing, in this paper we demonstrate that, in most cell lines, TSC2 localizes diffusely in the cytoplasm when conditions are optimal, whereas it relocalizes to lysosomes when any single inhibitory stimulus is applied to cells, thereby inhibiting mTORC1 (Fig. 7). In some cell lines, such as HeLa cells, TSC2 is constitutively lysosomal, even when amino acids and serum are present and no exogenous stress stimulus is applied, perhaps due to mutations they acquired during carcinogenesis or in culture. We confirm the important role of TSC2 localization in amino-acid signalling, and we provide data that suggest the universal nature of TSC2 localization in stress-induced mTORC1 inhibition.

## Methods

### Cell culture

All cell lines were grown at 37 °C, 5% CO_2_. Immortalized TSC2 WT (TSC2^+/+^p53^−/−^) and knockout (TSC2^−/−^p53^−/−^) MEFs, and embryonic kidney HEK293FT cells (Invitrogen), were cultured in high-glucose DMEM (#11965-092, Gibco), supplemented with 10% FBS from Biochrom. HeLa and HeLa-ATCC cervical adenocarcinoma cells, MCF-7 breast adenocarcinoma cells, NIH3T3 MEFs, Hepa1-6 mouse hepatoma cells, COS African green monkey (*C. aethiops*) kidney fibroblast-like cells and HepG2 human liver hepatocellular carcinoma cells were cultured in high-glucose DMEM containing 10% FBS from PAA. MCF-7 cells were also supplemented with 1 × non-essential amino acids (#11140-050, Gibco). All media were supplemented with 1 × Penicillin–Streptomycin (#15140-122, Gibco). TSC2^+/+^p53^−/−^, and TSC2^−/−^p53^−/−^ MEFs were a kind gift by David Kwiatkowski and Michael Hall, and were described elsewhere^62^. In addition to the batch of HeLa cells that we already possessed in the lab (kindly provided by Michael Boutros), an independent stock of HeLa cells was obtained from the ATCC repository (ATCC CCL-2) and cultured as described above.

The identity of HeLa, HEK293FT, MCF-7 and HepG2 cells was verified by the Multiplex human Cell line Authentication test (Multiplexion), which uses a single-nucleotide polymorphism-profiling approach and was performed as described at www.multiplexion.de. The HeLa, HEK293FT, MCF-7, HepG2, NIH3T3, Hepa1-6, TSC2^+/+^p53^−/−^ and TSC2^−/−^p53^−/−^ MEF cell lines were verified to be free of *Mycoplasma*, or contamination with cells of other species, according to the Multiplex cell Contamination Test Report (Multiplexion), as described at www.multiplexion.de.

### Cell treatments and media composition

Amino-acid starvation experiments were carried out as follows: for treatments in the presence of amino acids, cells were cultured in commercial, complete, amino-acid-containing, high-glucose DMEM media (#11965-092, Gibco). The respective custom-made −aa medium was formulated according to the Gibco recipe for high-glucose DMEM, omitting the amino acids, and filtered through a 0.22-μm filter device before use. All treatment media were supplemented with 10% dFBS. For this purpose, FBS was dialysed against PBS in 3,500 molecular weight cutoff (MWCO) dialysis tubing. Note that for these experiments the treatment strategy is specific for starvation of amino acids, whereas all other cell culture parameters remain unchanged.

For amino-acid starvation, normal media were replaced with media lacking amino acids for 1 h. For re-addition experiments, cells were first starved as described above for 1 h and then the starvation media were replaced with treatment media containing amino acids for 30 min. For serum starvation experiments, the culture media were replaced with media lacking FBS for 16 h. Insulin (final concentration 1 μM, #I9278, Sigma) was added to the indicated wells 15 min before lysis or fixation, whereas re-addition of FBS was for 30 min before lysis. For double starvations, cells were treated in serum-free DMEM for 16 h, and the media were replaced with serum- and amino-acid-free media 1 h before lysis or fixation.

Hyperosmotic stress conditions were applied by addition of concentrated (5 M) NaCl solution to the culture media to increase its concentration by 33–200 mM. The concentration of NaCl in serum-free normal culture media (high-glucose DMEM, Gibco) is 110.35 mM and the overall osmolality is 320–360 mOsm kg^−1^, according to the manufacturer's specifications. An increase of the NaCl concentration by 100 mM to full, serum-containing media raises osmolality to ca. 500 mOsm kg^−1^ (ref. 63).

Chemical and pharmacological treatments using Akt inhibitor VIII (#124018, Calbiochem), 2-DG (#D8375, Sigma) and CoCl_2_ (#60818, Sigma) were performed by adding the drugs directly to the medium at concentrations indicated in the figure legends. For the experiments in Supplementary Fig. 6, cells were pretreated with high- or low-glucose DMEM (Gibco) for 1 h and then the indicated amounts of 2-DG were added for another 30 min before lysis or fixation.

For experiments testing the effects of extracellular pH on mTORC1 activity and TSC2 localization, cells were treated with normal media containing FBS, which were pre-warmed in a humidified incubator at 37 °C, 5% CO_2_, before the pH was adjusted as indicated in the figures to 7.4–9.4. Cells were treated with the pH-adjusted media for 30 min before lysis or fixation. The pH of the media was checked post-treatment to ensure that it remains at the desired values during the course of the treatment. In contrast to NaHCO_3_-free DMEM media that are necessary for buffering pH values in the acidic range (for example, 5.4–6.4), normal NaHCO_3_-containing DMEM is capable of maintaining stable pH in the range of 7.4–9.4 for the time frame of our treatments.

Hypoxia was induced by incubating the cells for the indicated times in a hypoxic cell culture incubator (Heracell 150i, Thermo Scientific), at 37 °C, 5% CO_2_, where the O_2_ concentration was adjusted to 1%.

### Antibodies

Antibodies against phospho-S6K(T389) (#9205), S6K (#9202), phospho-4E-BP1(T37/46) (#9459), 4E-BP1 (#9452), p-Akt(T308) (#9275), p-Akt(S473) (#9271), Akt (#9272), p-AMPKα(T172) (#2535), AMPKα (#2532), p-p38(T180/Y182) (#9216), p38 (#9212), p-TSC2(T1462) (#3611), p-TSC2(S939) (#3615), TSC2 (#4308), TSC1 (#4906) and TBC1D7 (#14949) proteins were purchased from Cell Signaling Technology. An antibody against HIF-1α (GTX127309) was purchased from GeneTex. Antibodies detecting mouse LAMP2 (ABL-93) and human LAMP2 (A4B4) were obtained from Developmental Studies Hybridoma Bank. Monoclonal antibodies recognizing human and mouse α-tubulin (#T9026), FLAG-tag sequence (#F1804) and the peroxisomal marker PMP70 (#SAB4200181) were purchased from Sigma. All antibodies were used at 1:1,000 dilution for western blotting, except for the total TSC2, p-4E-BP1, total 4E-BP1 and total p38 antibodies that were used at 1:2,000. For immunofluorescence experiments, the TSC2 (validated in this study and in previous reports^4^,^18^), TSC1 (validated in this study), LAMP2 and PMP70 (validated in ref. 29) antibodies were used at 1:200 dilution.

### Plasmid constructs

The pcDNA3-FLAG-hRagA and hRagC expression vectors were described previously^4^. A similar vector expressing FLAG-tagged firefly luciferase was used as a negative control (pcDNA3-FLAG-Luc), and is described elsewhere^49^. The integrity of all constructs was verified by sequencing.

The pcDNA3-FLAG-LKB1 plasmid was a gift from Lewis Cantley (Addgene plasmid #8590), and has been described elsewhere^64^.

### Plasmid transfections

Plasmid DNA transfections in HEK293FT and HeLa cells were performed using Effectene (QIAGEN), according to the manufacturer's instructions.

### Cell imaging/immunofluorescence and confocal microscopy

For immunofluorescence experiments, cells were seeded on empty or fibronectin-coated glass coverslips and treated as indicated in each experiment. Following treatments, cells were fixed for 10 min at room temperature, with 4% paraformaldehyde (PFA) in PBS. Samples were washed/permeabilized twice with PBT solution (1 × PBS and 0.1% Tween-20) for 10 min, and blocked with BBT solution (1 × PBS, 0.1% Tween-20 and 0.1% BSA) for 45 min. Staining was performed with the indicated primary antibodies diluted in BBT (1:200) for 2 h, followed by four washes with BBT solution and 1 h incubation with appropriate highly cross-adsorbed secondary fluorescent antibodies (rabbit-FITC for TSC2 or TSC1, mouse- or rat-TRITC for LAMP2 and mouse-TRITC for PMP70). After two washes in PBT, nuclei were stained with DAPI (1:2,000 in PBT) and the coverslips were washed once more with PBT and mounted on slides using a glycerol-based mounting medium (80% glycerol, 1 × PBS and 0.4% propyl gallate). Images from single-channel captures are shown in greyscale. For the merged images, FITC is shown in green and TRITC in red. For co-localization experiments, representative magnified insets are shown on the right (top: TSC2 or TSC1; middle: LAMP2 or PMP70; bottom: merged). Images were captured using a × 40 objective lens and × 3 or × 5 digital zoom on an SP8 Leica confocal microscope.

All cell images within each panel were acquired and displayed using the same settings.

### Quantification of co-localization

Co-localization of proteins in confocal microscopy experiments was quantified using the Coloc2 plugin of Fiji software^65^. For each condition, 3–5 separate, representative confocal images were used, and Manders' co-localization coefficient (MCC) using automatic Costes thresholding^66^,^67^,^68^ was calculated for individual cells, excluding their nuclei to avoid false-positive co-localization, for a total of 20–40 individual cells per condition. The MCC value for each cell analysed, as well as the statistical analysis for each panel is provided in an accompanying table (Supplementary Data 1). MCC yields the fraction of the signal of interest (usually TSC2 or TSC1 in this study) that overlaps with a second signal (in our case lysosomes). For statistical analyses, the values represent mean and error bars represent s.e.m. Significance was calculated using analysis of variance (ANOVA), in the SigmaPlot 13.0 software. For Figs 1f, 3f, 4b and 5c and Supplementary Figs 7b,d, 12b and 13b, one-way ANOVA with *post hoc* Holm–Sidak comparisons was performed (pairwise comparisons to control). For Figs 1b,d, 2b,d,f,h and 6b,d and Supplementary Figs 4b,e,5b,e and 15e, one-way ANOVA with *post hoc* Holm–Sidak comparisons was performed (all pairwise comparisons). For Supplementary Fig. 6c, a two-way ANOVA with factors ‘Glucose level' and ‘2-DG' was performed. For the panels on Supplementary Figs 12d and 14d,f, unpaired Student's *t*-test was performed. No *post hoc* analysis was performed for Fig. 6d and Supplementary Figs 8b,15b and 16b,e, since ANOVA did not show any overall difference. One to three asterisks indicate *P*<0.05, *P*<0.01 and *P*<0.001, respectively, for the *post hoc* pairwise analyses. Statistically nonsignificant values (*P*>0.05) are indicated as ‘n.s.'. The exact *P* values for each comparison are provided in an accompanying table (Supplementary Data 2).

### Cell lysis and western blotting

For SDS–PAGE and immunoblotting experiments, cells were lysed in-well with ice-cold Triton lysis buffer (50 mM Tris (pH 7.5), 1% Triton X-100, 150 mM NaCl, 50 mM NaF, 2 mM Na-vanadate, 0.011 g ml^−1^ beta-glycerophosphate, 1 × PhosSTOP phosphatase inhibitors and 1 × Complete protease inhibitors) for 10 min on ice. Samples were clarified by centrifugation (15 min, 14,000 r.p.m., 4 °C), and SDS loading buffer was added to the soluble fraction before boiling. The samples were analysed by one-dimensional gel electrophoresis, and the presence of phospho- and total proteins was detected using the appropriate antibodies. The position of molecular weight markers (in kDa) is indicated on the right side of each immunoblot. To assist the interpretation of the immunoblots shown (Supplementary Fig. 10), quantification was performed using the LICOR Fc detection system and the ImageStudio software. Supplementary Figs 17 and 18 contain uncropped, full scans of western blot films, in the same orientation as in the corresponding cropped figures. For each uncropped panel, the figure panel numbers of the corresponding cropped figure are indicated.

### Co-immunoprecipitation

For co-immunoprecipitation experiments, cells were transfected with the indicated plasmids and lysed 24–48 h later in IP lysis buffer (50 mM Tris (pH 7.5), 1% Triton X-100, 150 mM NaCl, 50 mM NaF, 2 mM Na-vanadate, 0.011 g ml^−1^ beta-glycerophosphate, 1 × PhosSTOP phosphatase inhibitors and 1 × Complete protease inhibitors). FLAG-tagged proteins were incubated with 20 μl slurry of anti-FLAG M2 affinity gel (Sigma) for 2–3 h at 4 °C, washed with IP wash buffer (50 mM Tris (pH 7.5), 1% Triton X-100, 150 mM NaCl and 50 mM NaF) four times and beads were boiled in SDS loading buffer. Samples were analysed by one-dimensional gel electrophoresis and the presence of co-purifying proteins was detected using appropriate antibodies.

## Additional information

**How to cite this article:** Demetriades, C. *et al*. Lysosomal recruitment of TSC2 is a universal response to cellular stress. *Nat. Commun.* 7:10662 doi: 10.1038/ncomms10662 (2016).

## Supplementary Material

Supplementary Information Supplementary Figures 1-18

Supplementary Data 1 Quantification of IF [raw values represent Manders' colocalization coefficient (with automatic Costes threshold) for each cell analyzed]

Supplementary Data 2 Quantification of IF [p-values calculated based on raw values that represent Manders' colocalization coefficient (with automatic Costes threshold)]
