# Supplementary material for: Lysosomal recruitment of TSC2 is a universal response to cellular stress
Source: Nat Commun. 2016 Feb 12;7:10662. doi: 10.1038/ncomms10662 (PMC4754342; doi:10.1038/ncomms10662)
Supplement: Supplementary Information — Supplementary Figures 1-18 [file ncomms10662-s1.pdf]

Supplementary Figures

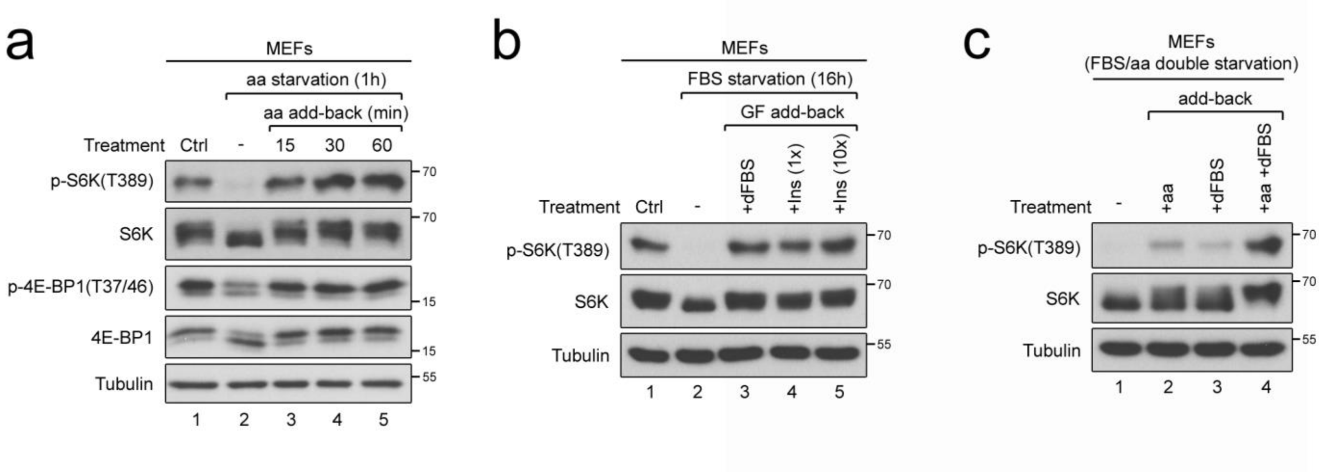

Supplementary Fig. 1: Related to main Fig. 1

(a) mTORC1 activity in MEFs responds as expected to the treatments performed in Fig. 1a. Cells were incubated with media containing (Ctrl) or lacking amino acids (-) for 1h. For re-addition samples, the aa starvation media were then replaced with aa-containing media for the indicated times. All treatment media contain dialyzed FBS.

(b) mTORC1 activity in MEFs responds as expected to the treatments performed in Fig. 1c. Cells were starved in medium lacking serum (FBS) for 16h and then left untreated (-) or treated with media containing dialyzed FBS (+dFBS) for 30 min or with the indicated amount of insulin (1x: 0.1 μM, 10x: 1 μM) for 15 min before lysis. Non-starved cells were used as control (Ctrl). Note that growth factor (GF, serum or insulin) re-addition restores mTORC1 activity, following over-night serum starvation.

(c) Amino acids and growth factors (FBS) cooperate to fully restore mTORC1 activity in starved MEFs. Cells were starved overnight for serum and for 1h of amino acids and then either left untreated (-) or amino acids (aa) and dialyzed FBS (dFBS) were re-added singly or in combination as indicated for 30 minutes before lysis. Note that re-addition of both aa and serum is necessary to fully restore mTORC1 activity in doubly-starved MEFs.

For all panels, representative blots of at least three independent biological replicates are shown.

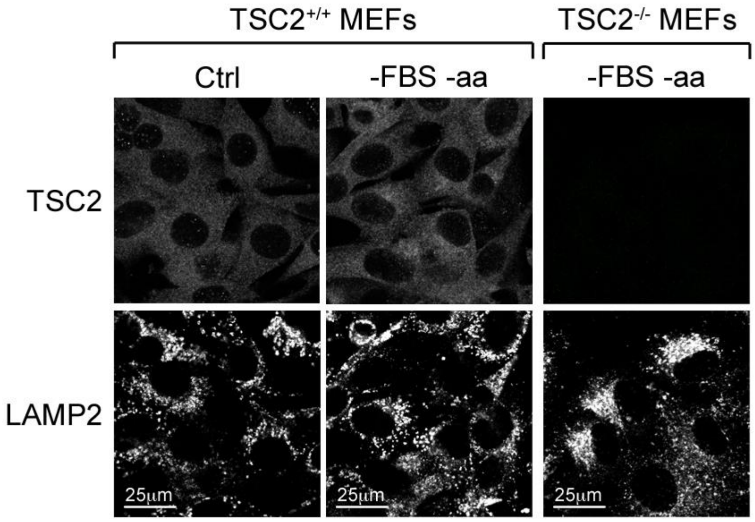

Supplementary Fig. 2: Related to main Fig. 1.

The α-TSC2 antibody used in this study recognizes endogenous TSC2 with high specificity. *TSC2* WT and *TSC2* knockout MEFs were left untreated (Ctrl) or starved for both serum and amino acids (-FBS -aa) before fixation as indicated. Endogenous TSC2 was detected by immunofluorescence. LAMP2 staining was used as a lysosomal marker and indicates the presence of cells. Note the absence of TSC2 signal in *TSC2*-null cells. Images of *TSC2* WT and *TSC2* knockout cells were taken with the same settings. (Representative of at least three biological replicates).

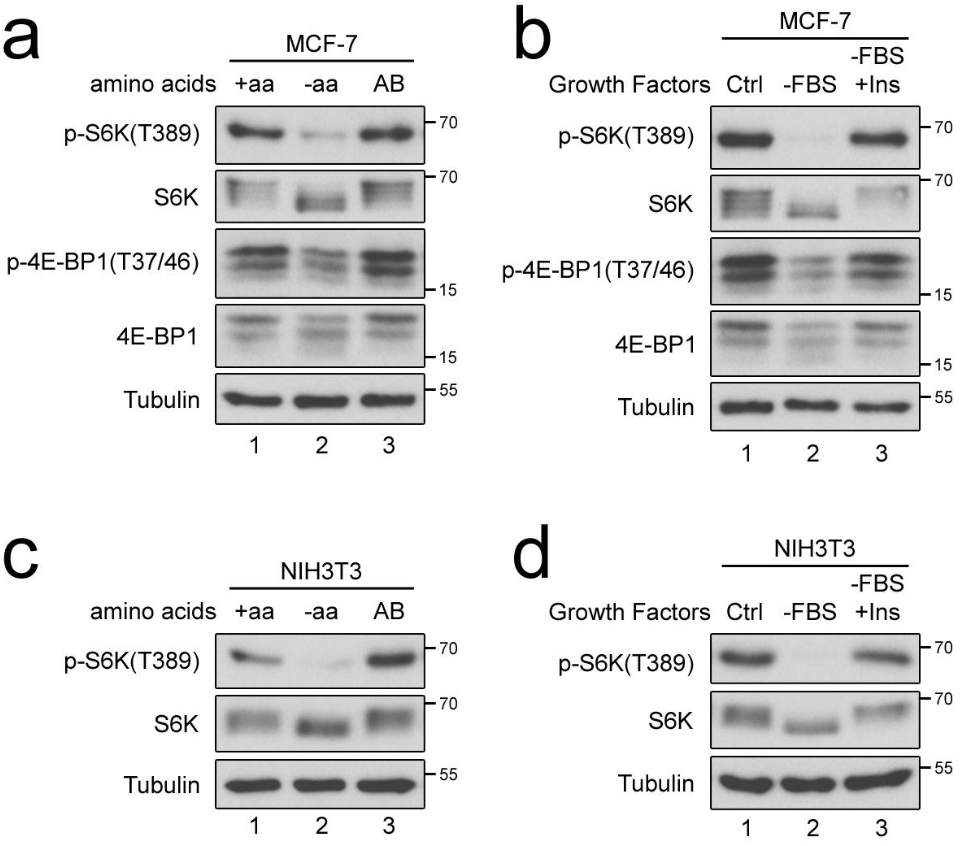

Supplementary Fig. 3: Related to main Fig. 2.

(a-d) mTORC1 activity responds as expected to changes in amino acid or growth factor signaling in MCF-7 (a-b) and NIH3T3 cells (c-d). Treatments were performed as in the respective panels in Fig. 2 and mTORC1 activity was analyzed by immunoblotting with the indicated antibodies. (Representative of two biological replicates).

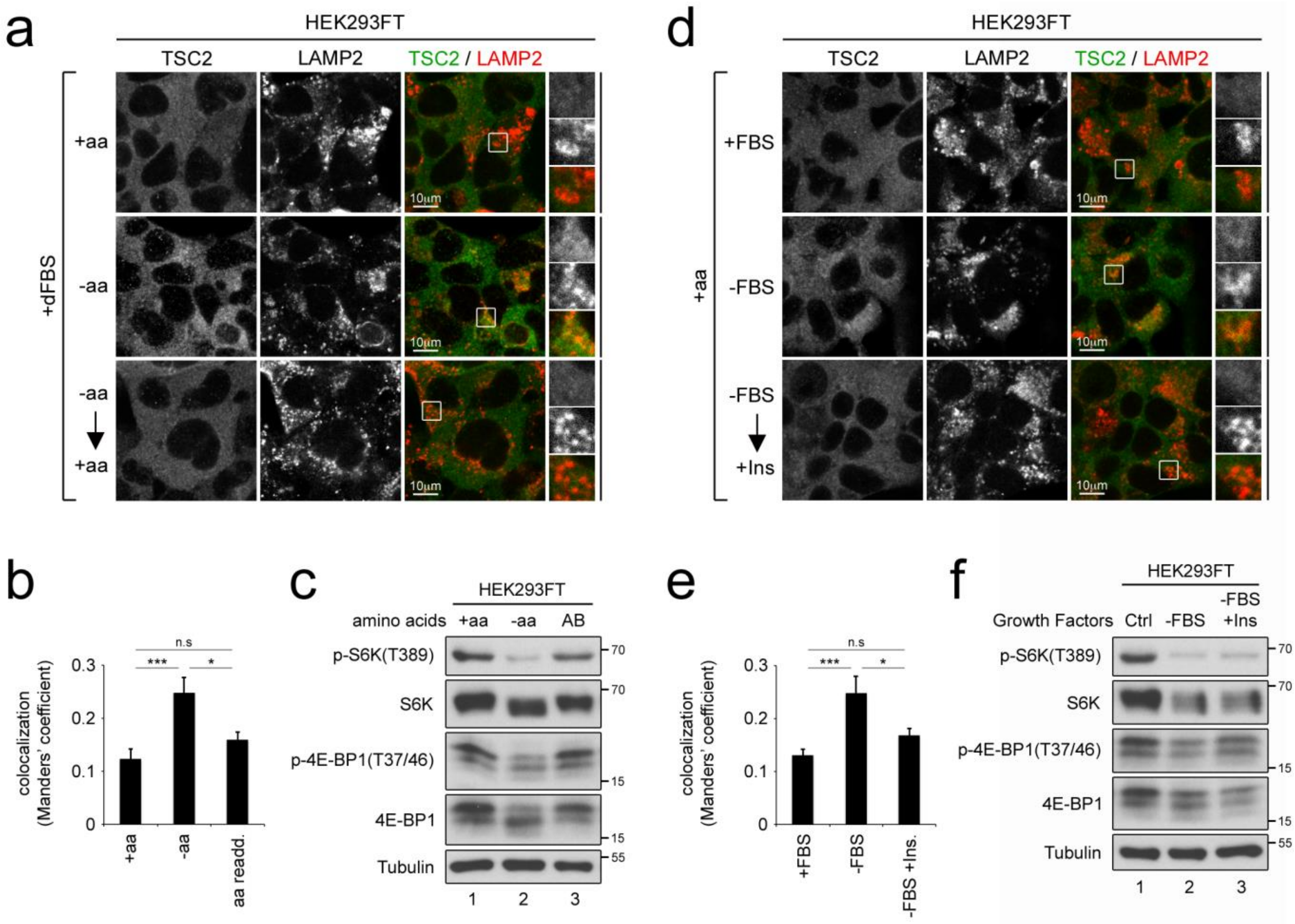

Supplementary Fig. 4: Related to main Fig. 2.

(a-b, d-e) Amino acid or serum starvation causes mild lysosomal accumulation of TSC2 in HEK293FT embryonic kidney cells. Treatments to assay the effects of amino acid starvation and re-addition (a) or the effects of serum starvation and insulin stimulation (d) on TSC2 localization in HEK293FT cells were performed as in main Figures 1a and 1c, respectively. TSC2 localization was analyzed as in Fig. 1a. The degree of colocalization between TSC2 and LAMP2 (automatically thresholded Manders' colocalization coefficient) is shown in (d, e) as mean ± SEM. *** $p<0.001$, * $p<0.05$, comparing samples as indicated, using one-way ANOVA.

(c, f) HEK293FT cells were treated as in (a, d) and mTORC1 activity was analyzed by immunoblotting with the indicated antibodies.

Data representative of three independent biological replicates are shown.

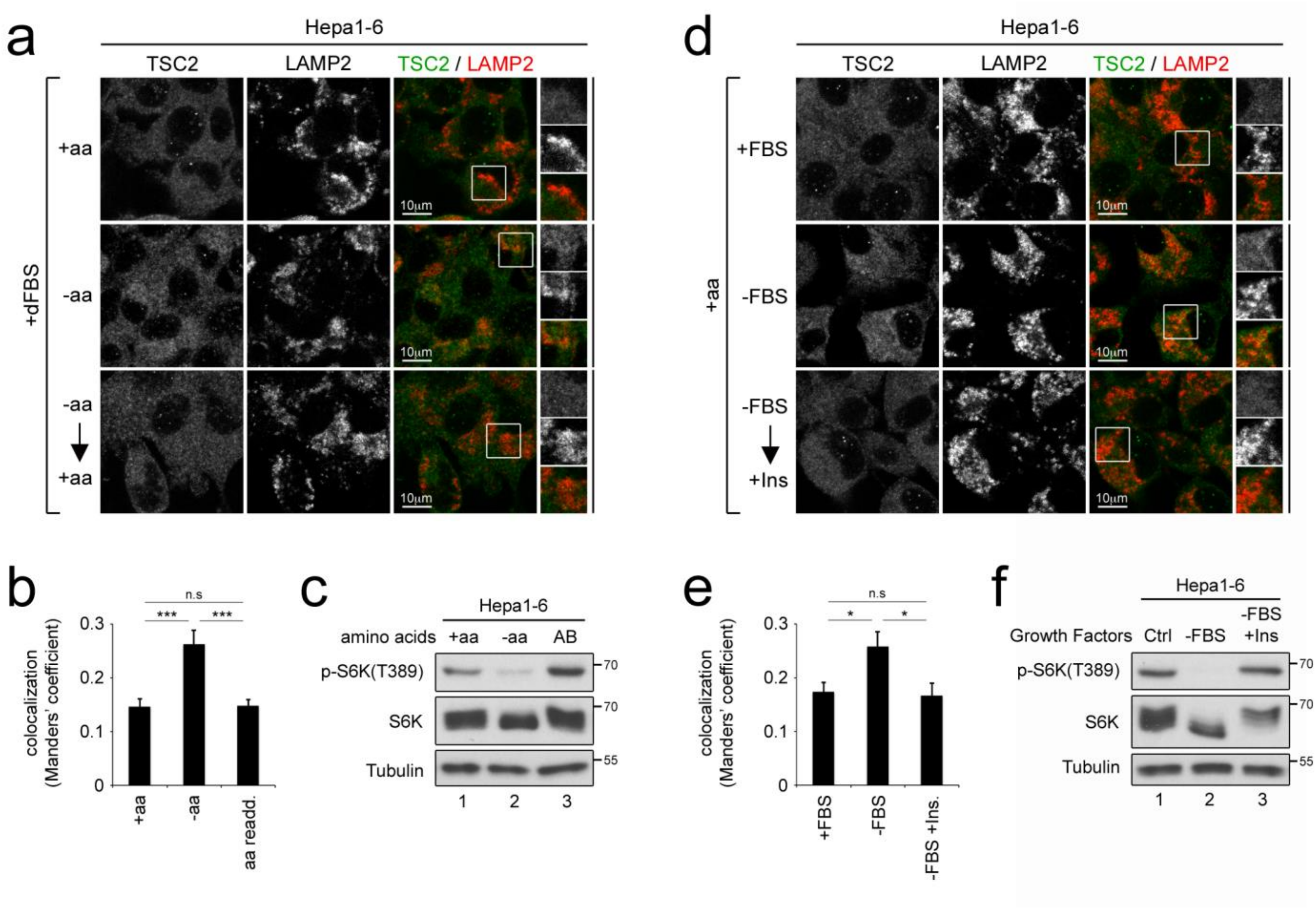

Supplementary Fig. 5: Related to main Fig. 2.

(a-b, d-e) Same as in Supplementary Fig. 4a-b, 4d-e using Hepa1-6 mouse hepatoma cells.

(c, f) Same as in Supplementary Fig. 4c, 4f using Hepa1-6 mouse hepatoma cells.

Data representative of two independent biological replicates are shown.

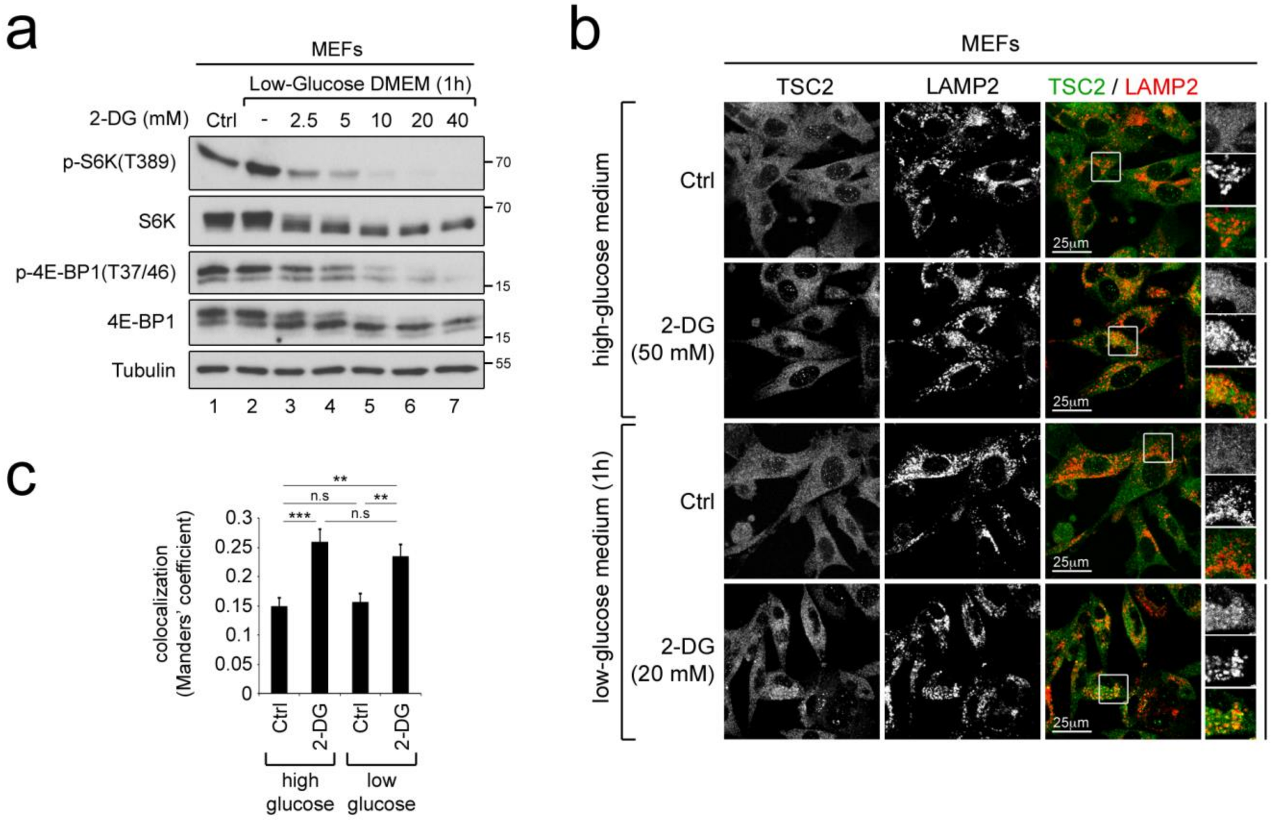

Supplementary Fig. 6: Related to main Fig. 3.

(a) Lower 2-DG concentrations are required to inhibit mTORC1 when cells are pre-treated with low-glucose medium, compared to cells in medium with high glucose levels (Fig. 3b). MEFs were incubated in high- (Ctrl) or low-glucose DMEM for 1h as indicated and then treated with the indicated amounts of 2-DG for 30 minutes before lysis. mTORC1 activity was analyzed by immunoblotting with the indicated antibodies. Note that approximately 2.5-fold less 2-DG causes a similar drop in mTORC1 activity (lanes 5-6), compared to main Fig. 3b (lanes 2-3).

(b-c) Lower 2-DG concentration is sufficient to drive lysosomal accumulation of TSC2 when cells are incubated in low-glucose medium. MEFs were treated as in (a) before fixation and TSC2 localization was analyzed by immunofluorescence. LAMP2 staining was used as a lysosomal marker.

Representative magnified insets are shown on the right (top: TSC2, middle: LAMP2, bottom: Merged) and colocalization between TSC2 and LAMP2 (automatically thresholded Manders' colocalization coefficient) is shown in (c) as mean ± SEM. *** $p<0.001$, ** $p<0.01$, comparing samples as indicated, using two-way ANOVA with factors "Glucose level" and "2-DG", and post-hoc one-way ANOVA for the pairwise analysis.

Note that 2.5-fold less 2-DG causes a similar accumulation of TSC2 on lysosomes when cells are pre-treated with low-glucose media.

Data representative of two biological replicates are shown.

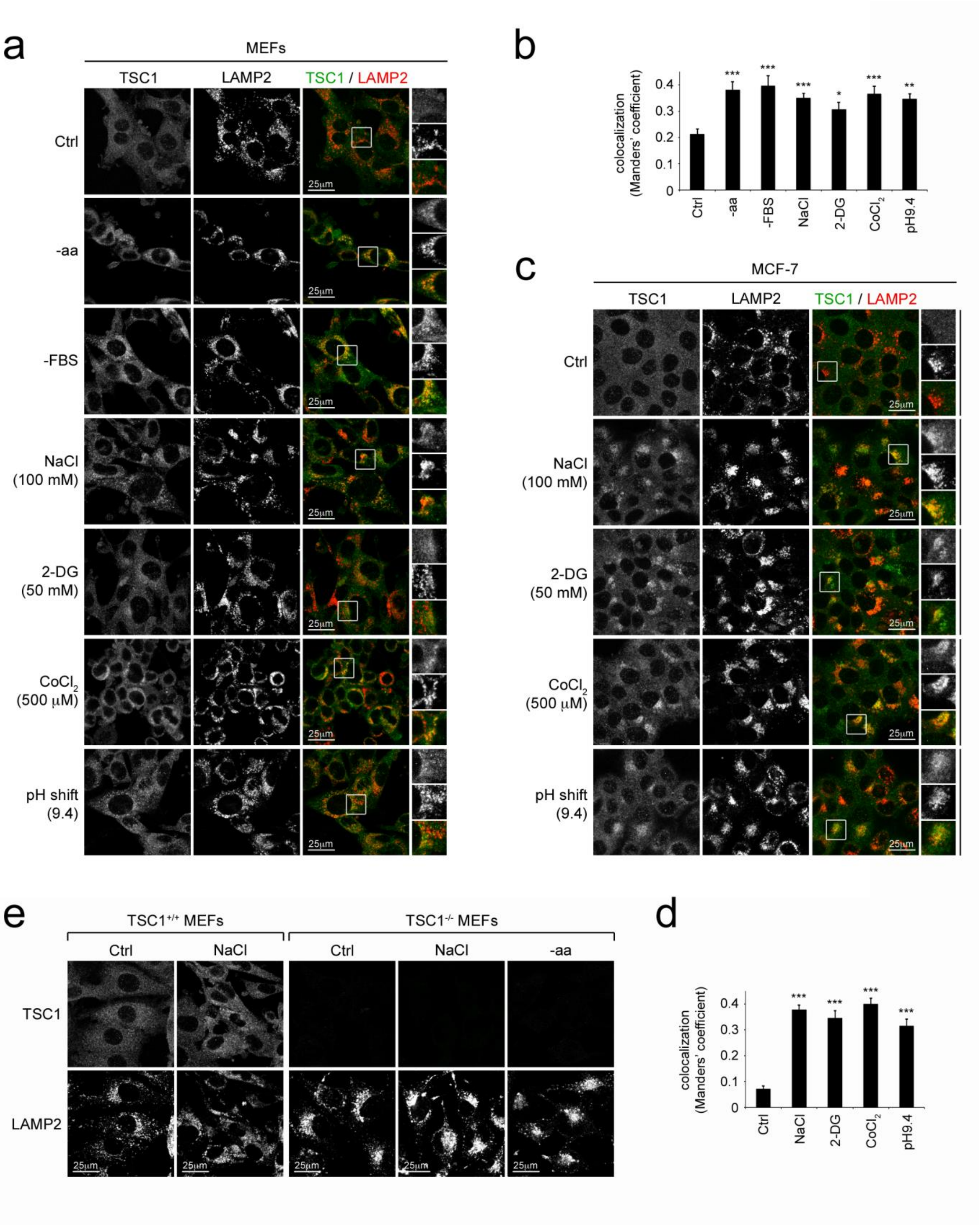

Supplementary Fig. 7: Related to main Figs. 3 and 4.

(a-b) TSC1 also relocalizes to lysosomes in response to cellular stress. MEFs were starved for amino acids or serum as in Figs. 1a and 1c, respectively, or

treated as in Fig. 3e to induce various stresses, and TSC1 localization was analyzed by immunofluorescence. LAMP2 staining was used as a lysosomal marker. Representative magnified insets are shown on the right (top: TSC1, middle: LAMP2, bottom: Merged) and colocalization between TSC1 and LAMP2 was quantified in (b) as in Fig. 3f (shown as mean ± SEM). Note that each individual inhibitory stimulus is sufficient to increase the lysosomal accumulation of TSC1, when applied singly to cells. *** $p<0.001$, ** $p<0.01$, * $p<0.05$ comparing each sample to "Ctrl", using one-way ANOVA. (Representative of two independent biological replicates).

(c-d) Same as in (a-b), using breast adenocarcinoma MCF-7 cells, treated as in Fig. 3e, and TSC1 localization was analyzed by immunofluorescence. LAMP2 staining was used as a lysosomal marker. Representative magnified insets are shown on the right (top: TSC1, middle: LAMP2, bottom: Merged), and colocalization is quantified in (d) as in (b) and shown as mean ± SEM. *** $p<0.001$ comparing each sample to "Ctrl", using one-way ANOVA. Note that each individual inhibitory stimulus is sufficient to cause lysosomal relocalization of TSC1, when applied singly to MCF-7 cells grown in serum- and amino-acid-replete media. (Representative of two independent biological replicates).

(c) The α-TSC1 antibody used in this study recognizes endogenous TSC1 with high specificity. *TSC1* WT ($TSC1^{+/+}$) and *TSC1* knockout MEFs ($TSC1^{-/-}$) were left untreated (Ctrl), treated with hyperosmotic media (NaCl) or starved for amino acids (-aa) before fixation as indicated. Endogenous TSC1 was detected by immunofluorescence. LAMP2 staining was used as a lysosomal

marker and indicates the presence of cells. Note the absence of TSC1 signal in *TSC1*-null cells. (Representative of two independent biological replicates).

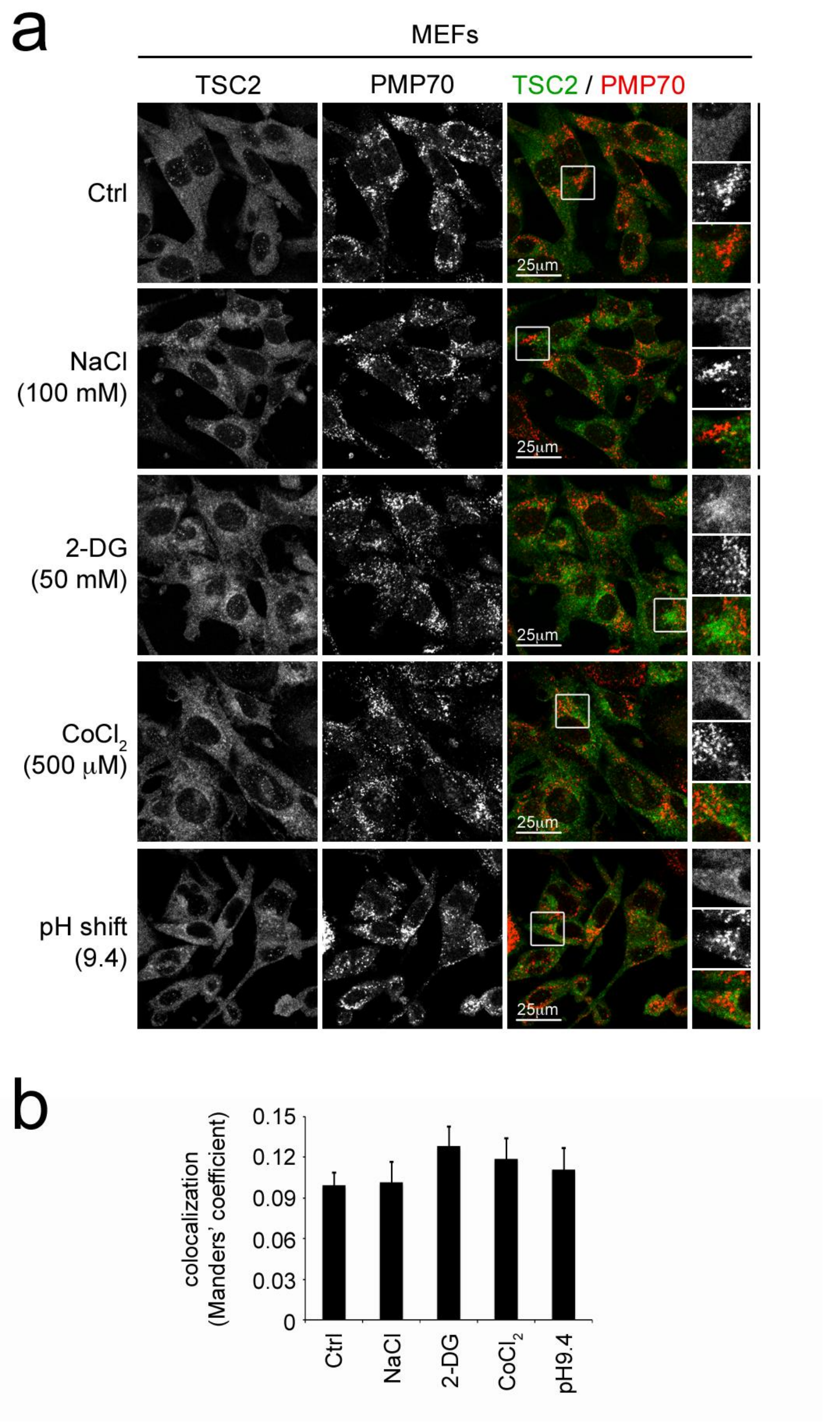

Supplementary Fig. 8: Related to main Fig. 3.

(a-b) TSC2 does not relocalize to peroxisomes in response to stress stimuli. MEFs were treated as in Fig. 3e and TSC2 localization was analyzed by immunofluorescence. PMP70 staining was used as a peroxisomal marker. Representative magnified insets are shown on the right (top: TSC2, middle:

PMP70, bottom: Merged) and colocalization between TSC2 and PMP70 was quantified in (b) as in Fig. 3f (shown as mean ± SEM, and analyzed using one-way ANOVA). Note the lack of colocalization between TSC2 and PMP70 in the presence or absence of stresses. (Representative of two biological replicates).

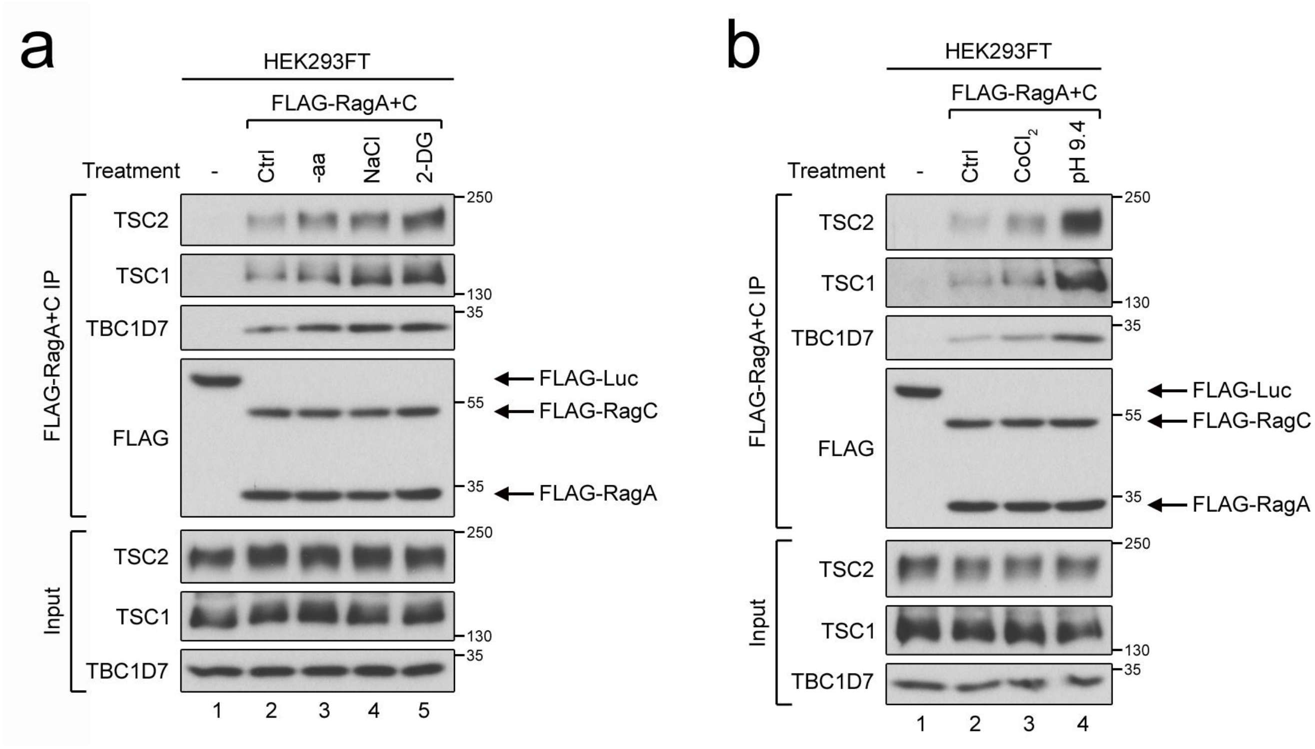

Supplementary Fig. 9: Related to main Fig. 3.

(a) Individual stresses increase binding between the TSC complex and the lysosomally localized Rag GTPases. HEK293FT cells were transfected with vectors expressing FLAG-tagged RagA and RagC or a control protein (FLAG-Luc) and then left untreated (Ctrl) or treated with aa-replete medium (-aa), hyperosmotic medium (NaCl, 100 mM) or 2-DG (50 mM) before lysis as indicated. The Rag GTPases were immunoprecipitated using FLAG affinity gel and the interaction with TSC2, TSC1, and TBC1D7 was analyzed using the indicated antibodies. Note that each stress stimulus, when applied individually to cells, causes an increase in binding between TSC2 and the RagA/C proteins, compared to cells treated with basal medium.

(b) Same as in (a), treating cells with cobalt chloride or with medium adjusted to pH 9.4.

For both panels, blots representative of at least two independent biological replicates are shown.

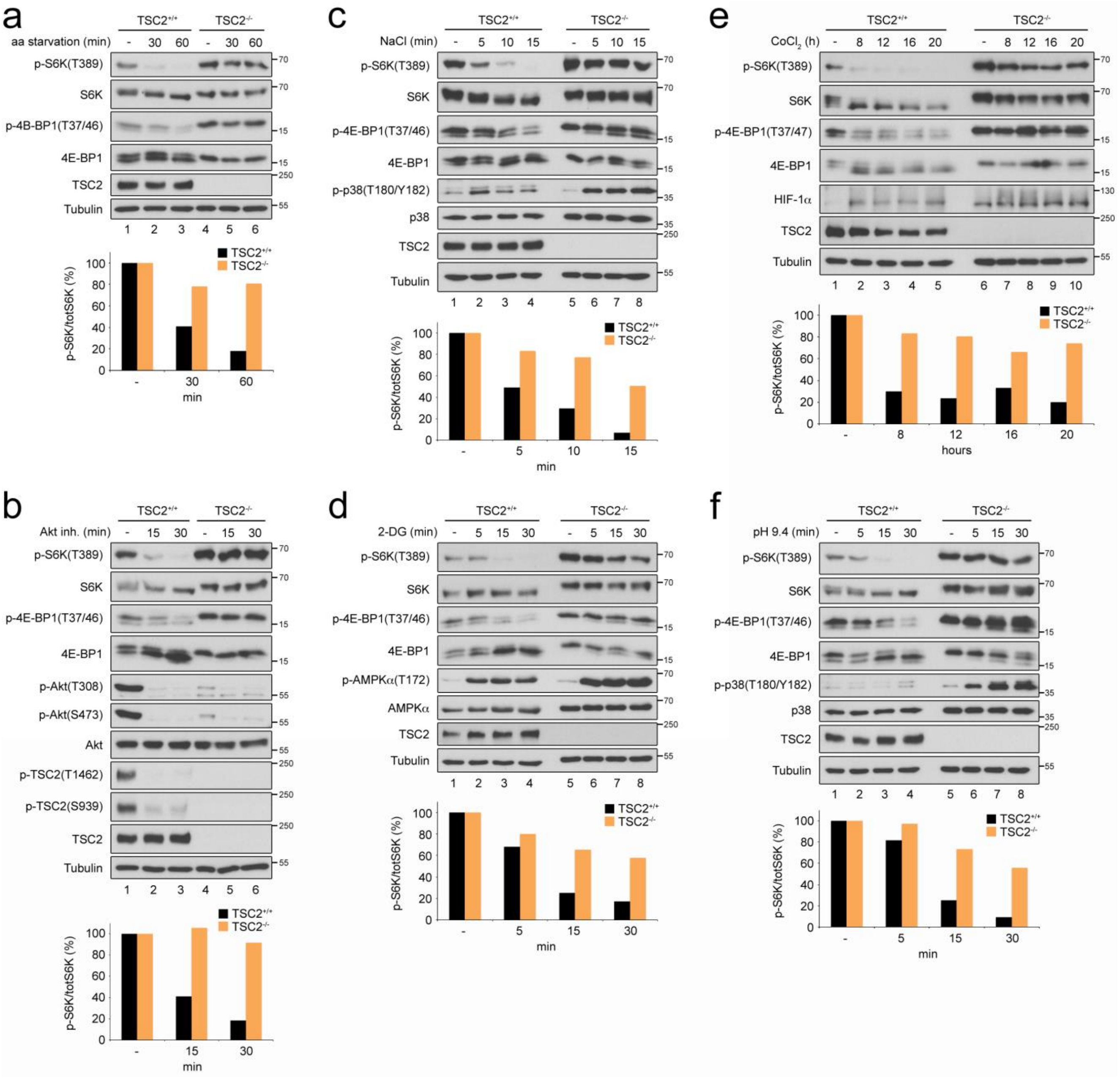

Supplementary Fig. 10: Related to main Fig. 3.

(a-f) mTORC1 inhibition in response to various stresses is mediated in part by TSC2. Wild-type (*TSC2*$^{+/+}$) or *TSC2*-null (*TSC2*$^{-/-}$) MEFs were treated with 100 mM NaCl (c), 50 mM 2-DG (d), 500 μM $CoCl_2$ (e) or medium with pH shifted at 9.4 (f) for the indicated times before lysis and mTORC1 activity was assayed by immunoblotting with the indicated antibodies. Phosphorylation of S6K was quantified using a LI-COR imaging system. To confirm the

previously described role of TSC2 in mediating part of the amino acid and Akt signals to mTORC1, WT or knockout cells were treated with media lacking amino acids (-aa) in the presence of dialyzed FBS (a), or with 10 μM Akt inhibitor VIII (b) for the indicated times. Note that for all treatments, mTORC1 activity drops robustly in WT MEFs, whereas *TSC2*-null cells retain significantly elevated levels of mTORC1 activity.

For all panels, blots representative of at least three independent biological replicates are shown.

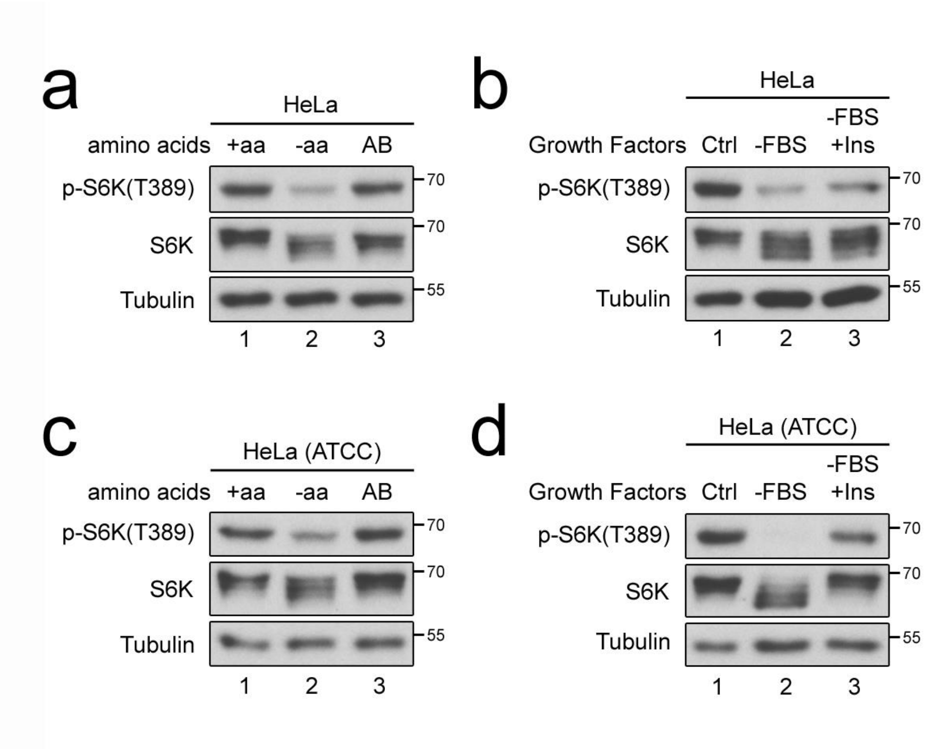

Supplementary Fig. 11: Related to main Fig. 6.

(a-b) mTORC1 activity in HeLa cells. Cells were treated as in Fig. 6a,c and mTORC1 activity was analyzed by immunoblotting with the indicated antibodies.

(c-d) Same as in (a-b), using HeLa (ATCC) cells.

For all panels, data representative of at least two biological replicates are shown.

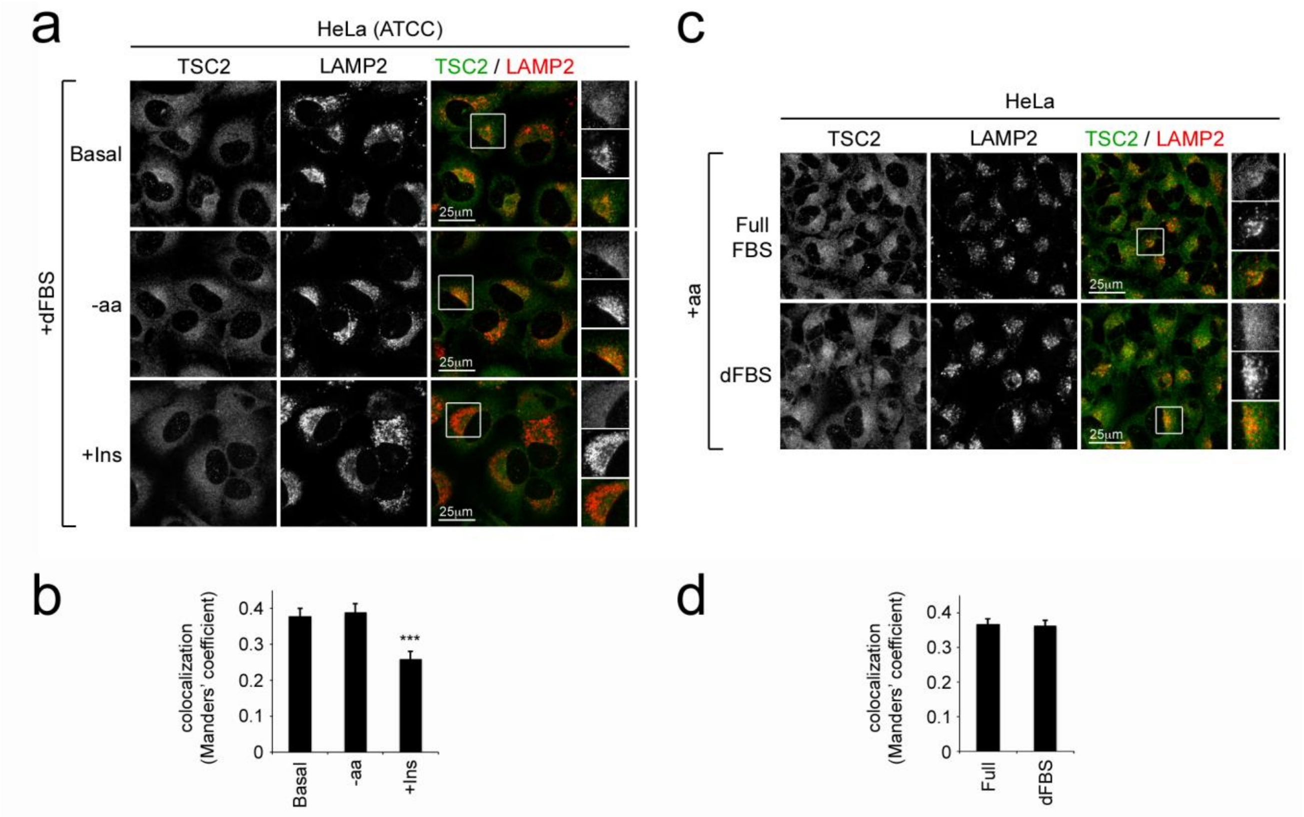

Supplementary Fig. 12: Related to main Fig. 6.

(a-b) The aberrant TSC2 localization of HeLa cells is not batch-specific. In addition to the stock of HeLa cells that we possess in the lab, an independent stock of HeLa cells was obtained from the ATCC repository (ATCC® CCL-2™) and TSC2 localization was assayed by immunostaining in basal conditions (Basal) as well as upon amino acid starvation (-aa) or insulin stimulation (+Ins). Colocalization between TSC2 and LAMP2 was quantified in (b) and shown as mean ± SEM. Similarly to the HeLa cell batch used in Fig. 6, TSC2 accumulates in LAMP2-positive areas even in control, serum- and amino acid-containing conditions, which are similarly present upon -aa and weaken significantly upon hyperstimulation with insulin (+Ins). *** $p<0.001$, compared

to “Basal”, using one-way ANOVA. (Representative of at least three independent biological replicates.)

(c-d) The aberrant TSC2 localization in HeLa cells is not caused by the use of dialyzed FBS. HeLa cells were left untreated (Full FBS) or the basal medium was replaced with medium containing dialyzed serum for 1h (dFBS) and TSC2 localization was assayed by immunostaining, using the indicated antibodies. Colocalization between TSC2 and LAMP2 was quantified and shown in (d) as mean ± SEM ($p=0.848$, using unpaired Student’s t-test). Note that TSC2 accumulates in LAMP2-positive areas both in the presence of normal and dialyzed FBS.

For all panels, data representative of two biological replicates are shown.

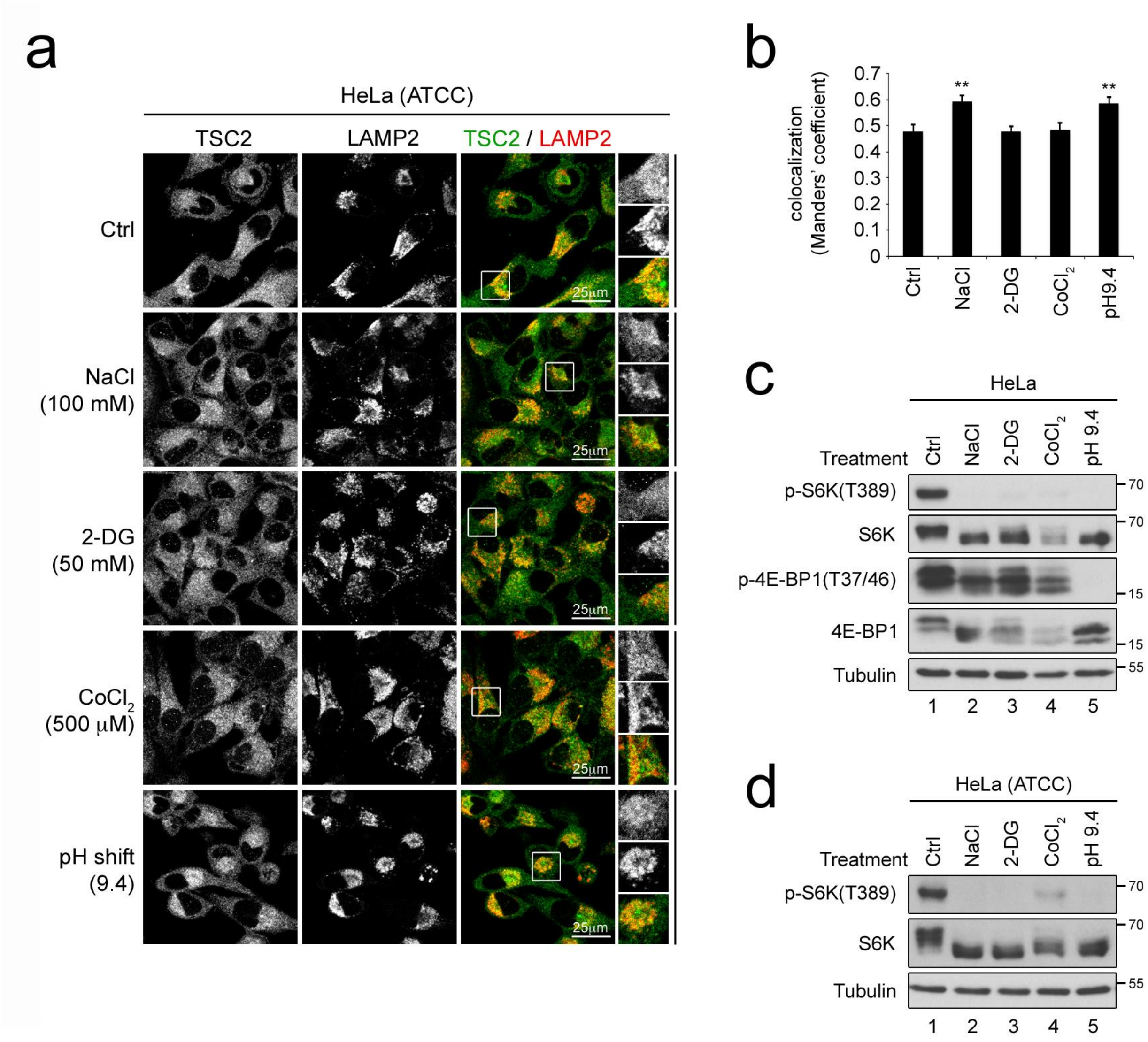

Supplementary Fig. 13: Related to main Fig. 6.

(a-b) HeLa cells show constitutive lysosomal localization of TSC2 in both control and stress conditions. HeLa (ATCC) cells were treated as in Fig. 3e and TSC2 localization was analyzed by immunofluorescence. LAMP2 staining was used as a lysosomal marker. Representative magnified insets are shown on the right (top: TSC2, middle: LAMP2, bottom: Merged) and colocalization between TSC2 and LAMP2 was quantified in (b) as in Fig. 3f (shown as mean ± SEM). ** $p<0.05$ compared to “Ctrl”, using one-way ANOVA.

(c) Individual stresses inactivate mTORC1 in HeLa cells. Cells were treated as in (a) and mTORC1 activity was analyzed by immunoblotting with the indicated antibodies.

(d) Same as in (c), using HeLa (ATCC) cells.

For all panels, data representative of at least two independent biological replicates are shown.

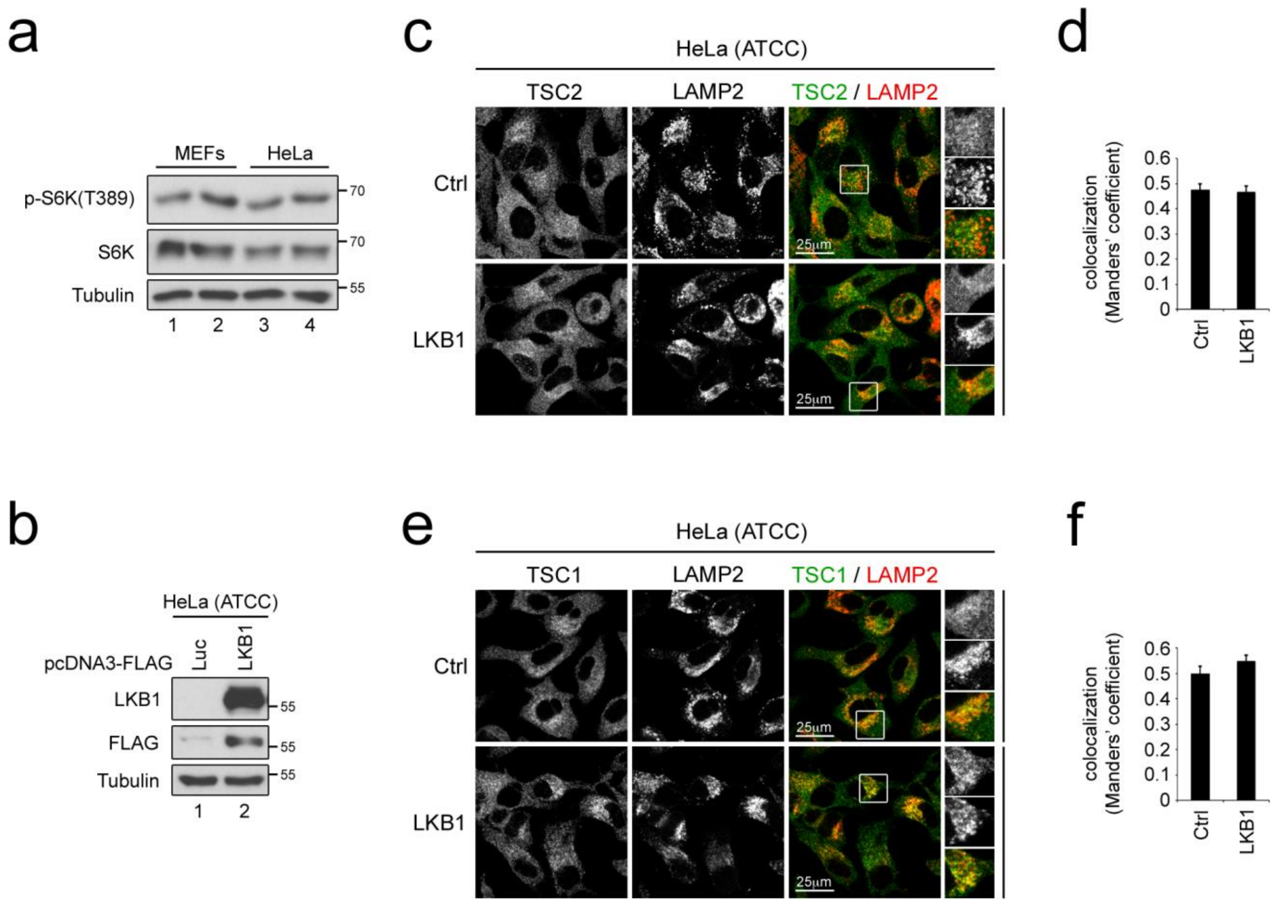

Supplementary Fig. 14: Related to main Fig. 6.

(a) HeLa cells show comparable mTORC1 activity to MEFs. Cells grown in complete medium were lysed and mTORC1 activity was analyzed by immunoblotting with the indicated antibodies.

(b-f) Exogenous expression of LKB1 does not rescue the constitutive lysosomal localization of TSC1/2 in HeLa cells. (b) Exogenous expression of LKB1 in HeLa (ATCC) cells confirmed by immunoblotting. Cells were transfected with a control (Luc) or an LKB1 expression vector, and the presence of FLAG-LKB1 was analyzed with the indicated antibodies. In LKB1-transfected cells, TSC2 (c-d) or TSC1 (e-f) localization was analyzed by immunostaining. LAMP2 staining was used as a lysosomal marker. Representative magnified insets are shown on the right (top: TSC2 (c) or

TSC1 (e), middle: LAMP2, bottom: Merged) and colocalization between TSC2 or TSC1 and LAMP2 was quantified and shown in (d, f) as mean ± SEM. p=0.772 and p=0.19 for (d) and (f), respectively, using unpaired Student's t-test. Note that TSC2 accumulates in LAMP2-positive areas even in LKB1 expressing HeLa cells, grown in the presence of serum and amino acids.

For all panels, data representative of two independent biological replicates are shown.

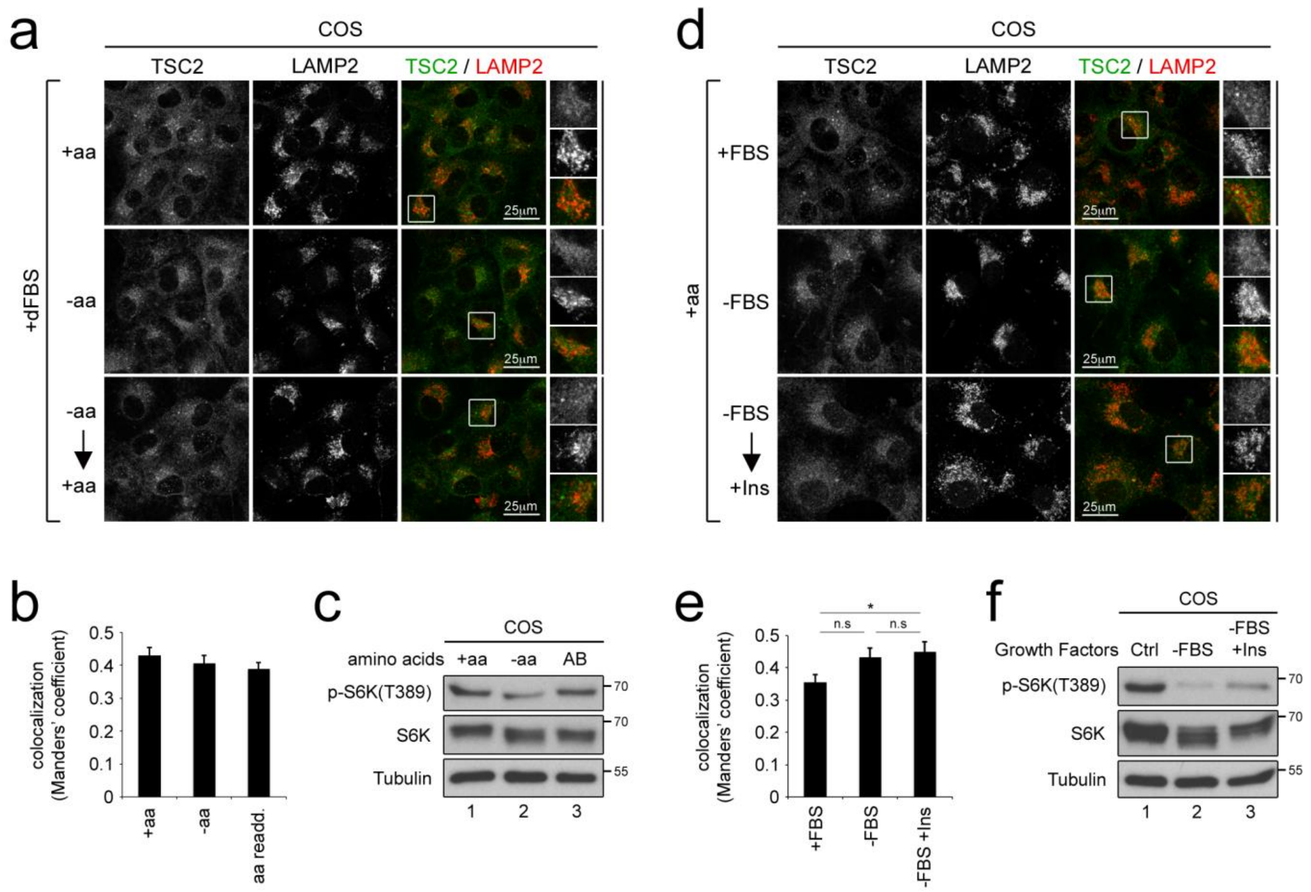

Supplementary Fig. 15: Related to main Fig. 6.

(a-b, d-e) Constitutive TSC2 accumulation in lysosomal regions in COS African green monkey (*Cercopithecus aethiops*) kidney fibroblast-like cells. Cells were treated and assayed, and colocalization was quantified as in Fig. 2a-d (shown in b, e as mean ± SEM). Note that TSC2 remains lysosomal even in the presence of amino acids and serum or upon insulin hyper-stimulation. * $p<0.05$ using one-way ANOVA.

(c, f) COS cells were treated as in (a, d) respectively and mTORC1 activity was analyzed by immunoblotting with the indicated antibodies. Note that mTORC1 in these cells does not respond well to amino acid starvation.

For all panels, data representative of two independent biological replicates are shown.

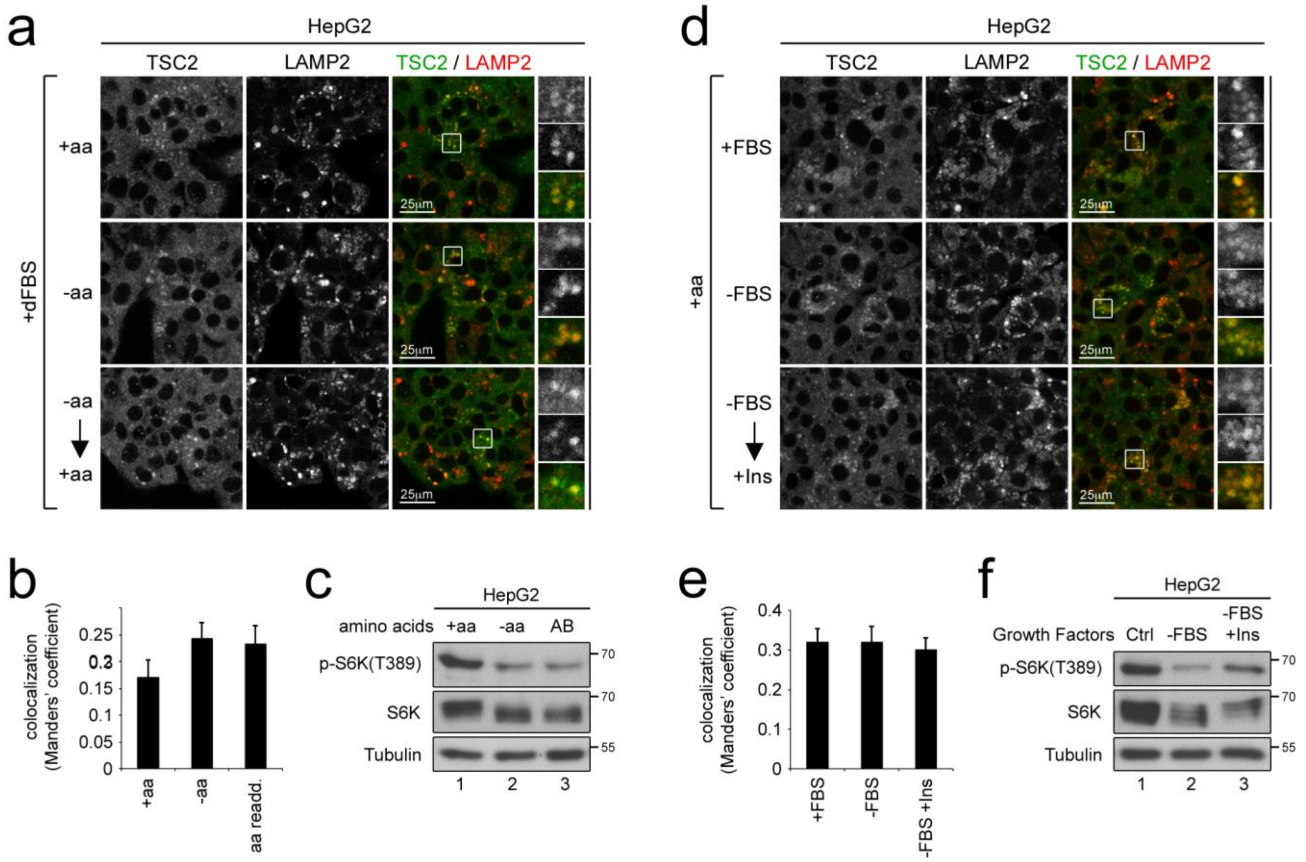

Supplementary Fig. 16: Related to main Fig. 6.

(a-f) Same as in Supplementary Fig. 15, using HepG2 human liver hepatocellular carcinoma cells. Note that TSC2 remains lysosomal even in the presence of amino acids and serum or upon insulin hyper-stimulation. Also note that mTORC1 in these cells does not respond well to amino acid re-addition, following starvation.

For all panels, data representative of two independent biological replicates are shown.

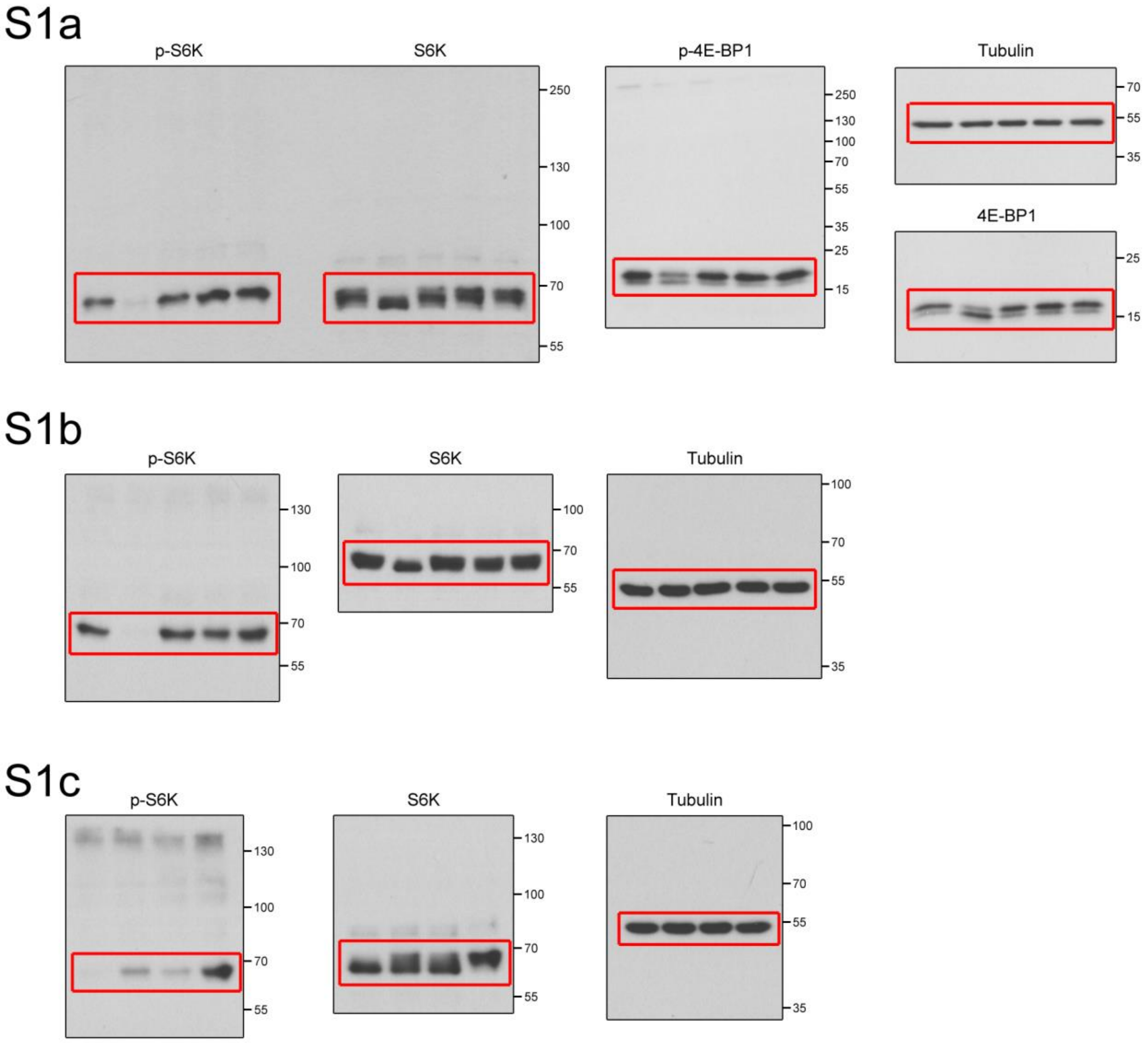

Supplementary Fig. 17: Uncropped western blots from Supplementary Fig. 1a-c.

S11a-b

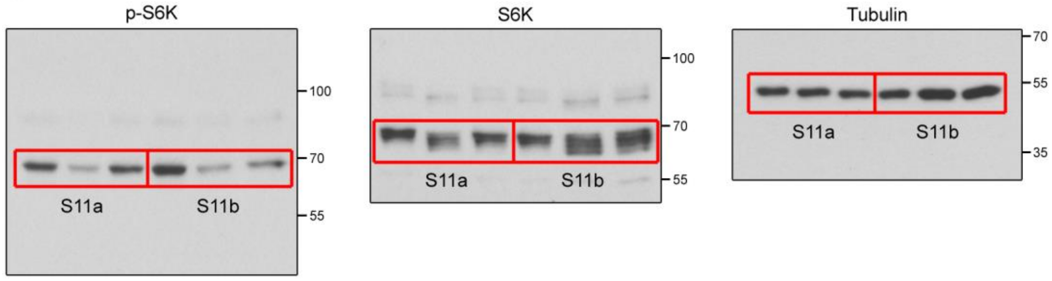

S11c-d

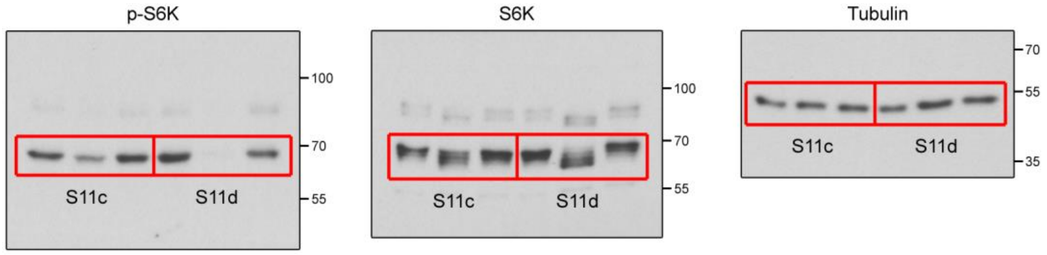

Supplementary Fig. 18: Uncropped western blot films from Supplementary Fig. 11a-d.
